# miR-375 prevents high-fat diet-induced insulin resistance and obesity by targeting the aryl hydrocarbon receptor and bacterial tryptophanase (*tnaA*) gene

**DOI:** 10.7150/thno.52558

**Published:** 2021-02-19

**Authors:** Anil Kumar, Yi Ren, Kumaran Sundaram, Jingyao Mu, Mukesh K Sriwastva, Gerald W Dryden, Chao Lei, Lifeng Zhang, Jun Yan, Xiang Zhang, Juw Won Park, Michael L Merchant, Yun Teng, Huang-Ge Zhang

**Affiliations:** 1 James Graham Brown Cancer Center, Department of Microbiology & Immunology, University of Louisville, KY 40202, USA.; 2 Department of Breast and Thyroid Surgery, The Affiliated Huaian No. 1 People's Hospital of Nanjing Medical University, Huaian, Jiangsu 223300, China.; 3 Department of Medicine, University of Louisville, Louisville, KY 40202, USA.; 4 Department of Pharmacology and Toxicology, University of Louisville, Louisville, KY 40202, USA.; 5 Department of Computer Engineering and Computer Science, University of Louisville, KY 40202, USA.; 6 KBRIN Bioinformatics Core, University of Louisville, Louisville, KY 40202, USA.; 7 Kidney Disease Program and Clinical Proteomics Center, University of Louisville, Louisville, KY, USA.; 8 Robley Rex Veterans Affairs Medical Center, Louisville, KY 40206, USA.

**Keywords:** Ginger derived nanoparticles, AhR, VAMP7, Exosomes, miR-375, *E. coli tryptophanase* (*tnaA*), indole, gut/liver axis, insulin resistance.

## Abstract

**Background:** Diet manipulation is the basis for prevention of obesity and diabetes. The molecular mechanisms that mediate the diet-based prevention of insulin resistance are not well understood. Here, as proof-of-concept, ginger-derived nanoparticles (GDNP) were used for studying molecular mechanisms underlying GDNP mediated prevention of high-fat diet induced insulin resistance.

**Methods:** Ginger-derived nanoparticles (GDNP) were isolated from ginger roots and administered orally to C57BL/6 high-fat diet mice. Fecal exosomes released from intestinal epithelial cells (IECs) of PBS or GDNP treated high-fat diet (HFD) fed mice were isolated by differential centrifugation. A micro-RNA (miRNA) polymerase chain reaction (PCR) array was used to profile the exosomal miRs and miRs of interest were further analyzed by quantitative real time (RT) PCR. miR-375 or antisense-miR375 was packed into nanoparticles made from the lipids extracted from GDNP. Nanoparticles was fluorescent labeled for monitoring their *in vivo* trafficking route after oral administration. The effect of these nanoparticles on glucose and insulin response of mice was determined by glucose and insulin tolerance tests.

**Results:** We report that HFD feeding increased the expression of AhR and inhibited the expression of miR-375 and VAMP7. Treatment with orally administered ginger-derived nanoparticles (GDNP) resulted in reversing HFD mediated inhibition of the expression of miR-375 and VAMP7. miR-375 knockout mice exhibited impaired glucose homeostasis and insulin resistance. Induction of intracellular miR-375 led to inhibition of the expression of AhR and VAMP7 mediated exporting of miR-375 into intestinal epithelial exosomes where they were taken up by gut bacteria and inhibited the production of the AhR ligand indole. Intestinal exosomes can also traffic to the liver and be taken up by hepatocytes, leading to miR-375 mediated inhibition of hepatic AhR over-expression and inducing the expression of genes associated with the hepatic insulin response. Altogether, GDNP prevents high-fat diet-induced insulin resistance by miR-375 mediated inhibition of the aryl hydrocarbon receptor mediated pathways over activated by HFD feeding.

**Conclusion:** Collectively our findings reveal that oral administration of GDNP to HFD mice improves host glucose tolerance and insulin response via regulating AhR expression by GDNP induced miR-375 and VAMP7.

## Introduction

Obesity is a complex and chronic disease that affects more than a one-third of the world's population 1. Changes in lifestyle, particularly increased consumption of unhealthy diets, are thought to be major causes of the current epidemic of obesity and type 2 diabetes (T2D). T2D is characterized by insulin resistance. Due to numerous dietary factors and dietary supplements that could contribute to modulating insulin signaling, it is challenging to identifying specific diet-derived factor(s) that contribute to modulating insulin signaling.

A number of diet-derived factors regulate the aryl hydrocarbon receptor (AhR) mediated signaling pathway which has been shown to regulate the insulin response 2. Mice that express a low-affinity AhR allele were less susceptible to obesity after exposure to a HFD and exhibited differences in fat mass, liver physiology and hepatocyte gene expression compared to mice with a high-affinity AhR 3. Serum AhR ligand levels were increased in samples from patients with T2D and were positively correlated with insulin resistance 4. However, the molecular mediators and mechanisms governing the association between diet, AhR and insulin pathway signaling in general are still elusive.

MicroRNAs (miRNAs) are a class of short, small regulatory RNAs that regulate the expression of specific target mRNA by binding to their 3' untranslated region (UTR) end 5. miRNAs are involved in many biological and metabolic processes including development, insulin resistance and adipocyte differentiation 6. miRNAs are also considered as biomarkers for several pathophysiological conditions/diseases such as type 1 and 2 diabetes, cancer and several other diseases 7-13. miR-375 was identified for the first time in abundantly expressed miRNA in pancreatic β-cells 14. Knockout of miR-375 in mice results in a hyperglycemic condition which reflected the insulin resistance condition 15, 16.

In this study, we use GDNP as a proof-of-concept to study the GDNP effect on gut epithelial AhR mediated signaling in mice fed a HFD. HFD-fed mice given GDNP via gavage showed an improved glucose tolerance and insulin response. Mechanistically, we found that GDNP treatment reversed a HFD mediated inhibition of the expression of miR-375. Additionally, the GDNP treatment altered the composition of the exosomal miRNA profile released from intestinal epithelial cells of HFD fed mice, with a concomitant significant increasing in exosomal miR-375. We further demonstrated that induced intracellular miR-375 leads to inhibition of the expression of AhR. Sorting of miR-375 into exosomes leads to inhibition of the production of the AhR ligand indole released from intestinal bacteria upon uptake of exosomes as well as increasing the insulin response in hepatocytes via downregulation of AhR.

## Methods

### Material and Reagents

#### Mice

6- to 8- week-old male C57BL/6 mice were purchased from the Jackson Laboratory (Bar Harbor, ME) and were maintained on a 12-h/12-h light/dark cycle in a pathogen-free animal facility at the University of Louisville. Animal care was performed following the Institute for Laboratory Animal Research (ILAR) guidelines, and all animal experiments were conducted in accordance with protocols approved by the University of Louisville Institutional Animal Care and Use Committee (Louisville, KY).

#### Human subjects

Fourteen healthy volunteers between the ages of 25 to 45 years (all males), seven obese (age matched) and fourteen patients with Type 2 diabetes (T2D) were recruited in the study. No healthy volunteers had a history of chronic gastrointestinal disease. Healthy volunteers and seven T2D patients were recruited from patients in the outpatient Endocrinology Clinic at University of Louisville Hospital, Louisville, Kentucky, USA. Seven obese and seven T2D patients were recruited in the Department of Surgery, Huai'an First People's Hospital, Huai'an, Jiangsu, China. Fecal and blood samples were collected from healthy, obese and T2D individuals. Type 2 diabetes was diagnosed according to the American Diabetes Association diagnostic criteria (American Diabetes Association 2012) in compliance with the declaration of the Helsinki principles. All participants were educated regarding their participation and signed a written consent form. Approval for the study was granted by the University of Louisville Research Ethics committee. Researchers were kept double blinded during the collection of data.

#### Cells

The murine colon cell line (MC-38) was purchased from American Type Culture Collection, ATCC and grown in tissue culture plates or dishes with Dulbecco Modified Eagle Medium (DMEM, Thermo Fisher Sci.) supplemented with 10% heat-inactivated fetal bovine serum (FBS), 100 mg/mL streptomycin, and 100 U/mL penicillin at 37 °C in a 5% CO_2_ atmosphere. Murine hepatocytes (FL83B) (American Type Culture Collection, ATCC) were grown in tissue culture plates with F12K medium (Thermo Fisher Sci) supplemented with 10% heat-inactivated fetal bovine serum (FBS), 100 U/mL penicillin, and 100 mg/mL streptomycin at 37 ºC in a 5% CO_2_ atmosphere.

### Generation of knock out cells

To generate VAMP7 knockout, we used a previously described protocol^17^ with minor modifications. In brief, cells were transfected with 1 μg of specific gene knockout plasmid (CRISPR/CAS9) using 30 μl of P3000 in 500 μl of Opti-MEM*®* in tube 1. Tube 2 contained 500 μl of Opti-MEM*®* plus 22 μl L3000 from the transfection kit (cat. no. L3000-015, Invitrogen, USA). The liquid from both tubes was mixed together and incubate at RT for 15 min. The mixture was added to a 100 mm dish of cells (50 - 60% confluent). The cells were incubated in culture medium for 72 h after the transfection, where upon GFP expression was checked. Knockout of targeted genes results were confirmed by qPCR and western blot. Plasmids were purchased from Santacruz Biotechnology (Table S6).

### Isolation and purification of ginger-derived nano-particles (GDNP)

Hawaiian ginger roots were purchased from a local market and the skin removed manually. The ginger root was chopped into small pieces, blended in a blender, the juice collected and diluted in PBS. The diluted ginger root juice was differentially centrifuged (500g for 10 min, 2,000g for 20 min, 5,000g for 30 min, 10,000g for 1 h) and the nano-particles then purified on a sucrose gradient (8, 30, 45 and 60% sucrose in 20 mM Tris-Cl, pH 7.2). The material settling out at the 30% sucrose band was collected and washed with PBS. Nanoparticle concentration and size distribution were determined using the nanoparticle tracking analysis method provided by the Malvern NanoSight NS300 (Malvern Instruments Ltd, Malvern, United Kingdom) 18. The purified GDNP was prepared for transmission electron microscopy (TEM) using a conventional procedure and observed using a FEI Tecnai F20 (EM facility, University of Alabama-Birmingham, Alabama, USA).

### High-fat diet mouse model

6 to 8-week-old C57BL/6 male mice (*n* = 10 per group) were fed either a regular chow diet (RCD; 10% Fat) or a high-fat diet (HFD; 60% Fat; detail description shown in Table S3). One HFD fed group was treated with PBS while a second HFD group received GDNP (6 x 10^8^ /mL) treatment in their drinking water for at least 12 months or their entire lifespan. The glucose and insulin tolerance test (GTT & ITT) were performed at 3, 6, 9 & 12 months after treatment. The data presented in the current manuscript is from a minimum of 12 months of treatment.

### miRNA PCR microarray

Total RNA containing small RNAs was isolated from tissues and cells using a miRNeasy mini kit (Qiagen; cat. no. 217004). miRNA expression profiling for exosomes was performed using the Qiagen miScript miRNA PCR Array Mouse miRBase Profiler (Cat# 331223) and an Applied Biosystems ViiA 7 Real-Time PCR System as described 17. Normalization to endogenous control genes included RNU6, SNORD61, SNORD68, SNORD72 and SNORD95 to correct for potential RNA input or RT efficiency biases. miRNA data generated from exosomes were comparatively analyzed by the online free data analysis software at https://dataanalysis.qiagen.com. Quantile normalization and subsequent data processing were performed and scatter plots representing differentially regulated miRNAs were generated.

### Affymetrix mRNA microarray

RNA was extracted from tissues using a Qiagen RNeasy mini kit (cat. no. 74104). 100 ng of RNA for each sample submitted to the Invitrogen/ThermoFisher Scientific Affymetrix facility, Santa Clara, CA, USA. Transcriptome Analysis Console (TAC) 4.0 from ThermoFisher Scientific was used to analyze the data.

### Quantitative reverse transcription polymerase chain reaction (qPCR) analysis mRNA expression

Total RNA was isolated from tissue and cells using a RNeasy mini kit (Qiagen). For analysis of *VAMP7*, *AhR*, and *IRS-1* & *2* mRNA expression, 1 μg of total RNA was reverse transcribed using SuperScript III reverse transcriptase (Invitrogen) and quantitation was performed using primers (Eurofins) with QuantiTect SYBR Green PCR (Qiagen). GAPDH was used for normalization. The primer sequences are listed in Table S4. qPCR was run using the BioRad CFX96 qPCR System with each reaction run in triplicate.

### TargetScan and BLASTN analysis

The online available TragetScan tool was used for predicting the putative target site of miR-375-3p (WWW.targetscan.org).

The Basic Local Alignment Search Tool was used for sequence matching. The link is provided below.

https://blast.ncbi.nlm.nih.gov/Blast.cgi?PROGRAM=blastn&PAGE_TYPE=BlastSearch&LINK_LOC=blasthome.

### Biodistribution targeting of orally administrated H-Exo by live imaging and confocal microscopy

Six hours after oral administration of 2 × 10^12^ /dose/mouse of either PKH26 or DiR fluorescent dye (Sigma) labelled GDNP, mice were sacrificed and the small intestine, colon, MLN, spleen and liver tissues were imaged. DiR fluorescent signal was detected and measured using the Imaging Station Pearl Impulse (*Li-COR* Biosciences). The labeled GDNP in the gut of mice were visualized using an Odyssey CLx Imaging System (*Li-COR* Biosciences). PKH26 signal in frozen tissue sections was observed using a confocal laser scanning microscopy system (Nikon, Melville, NY). The method has been described previously 19.

### Isolation and purification of feces exosomes

Fresh fecal pellets were re-suspended in PBS and minced manually. Differential centrifugation was employed to isolate the fecal exosomes. Fecal suspensions were centrifuged at 1000g for 10 min, 2,000g for 20 min and 4,000g for 30 min to remove large particles. The supernatants were centrifuged at 8,000g for 1 h to remove micro-particles. Finally, each suspension was centrifuged at 100,000g for 2 h. Pellets were suspended in PBS. The exosomes were further purified by sucrose gradient (8, 30, 45 and 60% sucrose in 20 mM Tris-Cl, pH 7.2) centrifugation. An aliquot of the purified exosomes was fixed in 2% paraformaldehyde for transmission electron microscopy (EM) using a conventional procedure and observed using an FEI Tecnai F20 (EM facility at the University of Alabama-Birmingham, Alabama, USA) 19, 20. The EM was done using the following settings, 80 kV at a magnification of 15,000 and defocus of 100 and 500 nm.

### Nanoparticle tracking analysis

Sucrose purified nanoparticle (H-Exo) samples were analyzed for particle concentration and size distribution using the nanoparticle tracking analysis method provided by the Malvern NanoSight NS300 (Malvern Instruments Ltd, Malvern, United Kingdom) 18. Measurements were performed in accordance with the manufacturer's instructions. Briefly, for the NanoSight300, three independent replicates of diluted particle preparations in PBS were injected at a constant rate into the tracking chamber using the provided syringe pump. The samples were tracked at room temperature for 60s. Shutter and gain were manually adjusted for optimal detection and were kept at optimized settings for all samples. The data were captured and analyzed with NTA Build 127 software (version 2.2, Malvern Instruments Ltd, Malvern, UK).

### Lipid extraction from GDNP

Total lipids were extracted from sucrose purified and washed bands of processed ginger-derived nano-particles 19. Briefly, 1.9 mL 2:1 (v/v) methanol:chloroform was added to 0.5 mL of GDNP in PBS and 0.625 mL of chloroform and water were added sequentially and the mixture vortexed thoroughly. The aqueous and organic phase were separated by centrifugation at 2,000 r.p.m. for 10 min at 22 °C in glass tubes. The organic phase was collected using a glass pipette and dispensed into fresh glass tubes. The organic phase was dried under nitrogen (2 psi). Total lipids were determined using the phosphate assay.

### Nanoparticle preparation from lipid extracted from GDNP

To prepare GDNP nanoparticles, GDNP-derived lipids were extracted with chloroform and dried under vacuum 20. 300 nmol of lipid was suspended in 600 μl of 155 mM NaCl with or without microRNA (miR-375), antisense-miR375 or scramble RNA (20 nm each). 4 μl of PEI was added. The mixture was ultra-sonicated for 20 minutes at 4 °C in a bath sonicator (FS60 bath sonicator, Fisher Scientific, Pittsburg, PA). After sonication, nanoparticles were pelleted by ultracentrifuge for 1 h at 100,000g. Before being used in experiments, the nanoparticles were homogenized by passing the samples through a high-pressure homogenizer (Avestin Inc., Ottawa, Canada) using a protocol provided in the homogenizer instruction manual.

### Pull down assay

MC-38 cells were transfected with 500 ng of biotinylated miR-375 or scrambled RNA using RNAiMAX transfection reagent (ThermoFisher). After 24 hours of transfection, cells were harvested, washed with ice-cold PBS. Cells were lysed by RIPA buffer supplemented with a protease inhibitor cocktail and incubated on ice for 30 min. The lysate was centrifuged at 20,000g for 15 min. The supernatant was mixed with pre-washed streptavidin beads and incubated on ice for 1 h. Tubes containing beads were exposed to a magnetic platform and the supernatant was carefully removed with a pipette. Beads were washed three times and products were eluted with elution buffer (4 mg/mL biotin in 25 mM Tris-HCl, 250 mM NaCl pH8.5).

### Labelling of nanoparticles

Nanoparticles were labeled with DIR or using the PKH26 Fluorescent Cell Linker Kits (Sigma) and following the manufacturer's instructions. Nanoparticles were suspended in 250 μl of diluent C with 4 μl of DIR or PKH26 dye and subsequently incubated for 30 min at room temperature 20. After washing with PBS and centrifugation at 10,000g for 1 h at 4 °C, the pellet was washed twice to remove unbound dye and finally re-suspended in PBS and used in experiments.

### Glucose and Insulin tolerance tests (GTT & ITT)

For glucose tolerance tests, after an overnight fast, baseline glucose levels were determined using a glucometer (Priology, USA). Then, mice were given an intraperitoneal injection of glucose (dextrose) at a dose of 2 mg/g of body weight 21. The blood glucose levels were measured at 30, 60, 90, and 120 min after glucose injection. For insulin tolerance tests, mice were fasted for 6 h and basal blood glucose levels were determined. Mice were then given an intraperitoneal injection of insulin (1.2 units/g of body weight). The blood glucose levels were measured at 30, 60, and 90 min (otherwise indicated in figures) after insulin injection.

### Western blot analysis

The tissues were washed with ice-cold PBS and homogenized. The cells were lysed in radio-immunoprecipitation assay (RIPA) lysis buffer with the addition of protease inhibitor for 1 h at 4 °C. The crude lysates were centrifuged at 14,000g for 15 min. Protein concentrations were determined using the BioRad Protein Assay Reagent. Samples were diluted in 1x SDS sample buffer. Proteins were separated on a 10-12% or gradient SDS-PAGE and transferred to nitrocellulose membranes (Bio-Rad). Individual protein was detected with specific antibodies and visualized by infrared fluorescent secondary antibodies (Table S5). The protein bands were visualized and analyzed on an Odyssey CLx Imager (LiCor Inc, Lincoln, NE).

### Flow cytometry

Mice liver were harvested after perfusion (Ca^2+^-Mg^2+^ free HBSS containing 0.5 mM EGTA, 10 mM HEPES and 4.2 mM NaHCO_3_ supplemented with Type I collagenase (0.05%) and trypsin inhibitor (50 μg/mL; pH 7.2)) and then transferred into complete medium as described elsewhere 19. Cells isolated from liver tissue were fixed with 2% paraformaldehyde (PFA) and stained with albumin and F4/80 fluorochrome conjugated antibodies for 45 min at 4 °C. Stained liver cells (monocytes and hepatocytes) treated with PKH26^+^ nanoparticle or fecal exosomes were acquired using a BD FACSCanto flow cytometer (BD Biosciences, San Jose, CA) and analyzed using FlowJo software (Tree Star Inc., Ashland, OR).

For sorting of bacteria, fecal samples from mice gavaged with PKH-26 labeled exosomes (after 6 h) were re-suspended in PBS and centrifuged at 2000g for 10 min to remove large particles. Suspensions from this step were used for sorting. PKH-26 positive bacteria were sorted using a BD FACSAria^TM^ III instrument equipped with 488 and 633 nm laser.

### Confocal microscopy

For frozen tissue sections, periodate-lysine-paraformaldehyde (PLP) fixed tissues were dehydrated with 30% sucrose in PBS overnight at 4 °C and embedded into optimal cutting temperature (OCT) compound. Tissues were subsequently cut into ultrathin slices (5 μm) using a microtome. The tissue sections were blocked with 5% bovine serum albumin (BSA) in PBS. Primary antibodies (1:800) were added and incubated at 4 °C overnight. Sections were washed three times followed by secondary antibodies conjugated to a fluorescent dye (at 1:2000 dilution). Nuclei were stained with 4', 6-diamidino- 2-phenylindole dihydrochloride (DAPI). For *in-vitro* cultured cells, 2 × 10^5^ cells were grown on coverslips in six well plates and co-cultured with PKH-26 labeled fecal exosomes for 16 h at 37 °C in a CO_2_ incubator. Cells were washed with PBS and fixed with 2% PFA. Nuclei were stained with DAPI. Tissues and cells were visualized via confocal laser scanning microscopy (Nikon, Melville, NY).

### Insulin signaling array of hepatocytes cultured with Nano-miR375

FL83B (0.3 × 10^6^ per well) cells were seeded into a six well plate containing DMEM/F12 medium supplemented with 10% FBS. After achieving 50-60% confluence, nanoparticles (2 × 10^6^ /mL) packed with miR-375 or scramble miR were added and incubated for 12 h at 37°C in a 5% CO_2_ atmosphere. Cells were washed with PBS and processed for RNA isolation or protein extraction for western blots. 1 μg of total RNA was reverse transcribed using Superscript III reverse transcriptase (Invitrogen). The insulin signaling array (PAMM030ZE) from Qiagen was performed using an Applied Biosystems ViiA^TM^ 7 Real-Time PCR System in accordance with manufacturer's instructions.

### Indole estimation

Indole levels in mice or human feces and plasma were estimated using the Quantichrom^TM^ Indole Assay kit (DIND- 100) from BioAssay Systems in accordance with manufacturer's instructions. Briefly, 100 μl of standards or samples were placed into separate wells (triplicates) of a clear flat bottom 96-well plate. 100 μl of reagent was added to each well. The plate was gently tapped to mix. The optical density was measured at 565 nm.

### Lipids analysis in plasma

Peripheral blood samples of mice were collected into non-heparinized capillary tubes coated with 4% sodium citrate. Plasma was harvested by centrifugation at 2000 g for 15 min. The levels of cholesterol and triglycerides were determined using a Piccolo lipid panel plus (Abaxis Inc, USA).

### LC-MS analysis of plasma and fecal metabolites

Exosome-free fecal supernates and plasma samples from lean, PBS and GDNP HFD mice were used for LC-MS analysis. All samples were analyzed using a Thermo Q Exactive HF Hybrid Quadrupole-Orbitrap Mass Spectrometer coupled with a Thermo DIONEX UltiMate 3000 HPLC system (Thermo Fisher Scientific, Waltham, MA, USA). The UltiMate 3000 HPLC system was equipped with a hydrophilic interaction chromatography (HILIC) column and a reverse phase chromatography (RPC) column. The HILIC column was a SeQuant® ZIC®-cHILIC HPLC column (150 × 2.1 mm i.d., 3 µm) purchased from Phenomenex (Torrance, CA, USA). The RPC column was an ACQUITY UPLC HSS T3 column (150 × 2.1 mm i.d., 1.8 µm) purchased from Waters (Milford, MA, USA). The two columns were configured in parallel 2DLC mode 22.

For 2DLC separation, the mobile phase A for RPC was water with 0.1% formic acid and the mobile phase A for HILIC was 10 mM ammonium acetate (pH adjusted to 3.25 with acetate). Both RPC and HILIC used the same mobile phase B, acetonitrile with 0.1% formic acid. The RPC gradient was 0 min, 5% B, hold for 5.0 min; 5.0 min to 6.1 min, 5% B to 15% B; 6.1 min to 10.0 min, 15% B to 60% B, hold for 2.0 min; 12.0 min to 14.0 min, 60% B to 100% B, hold for 13.0 min; 27.0 min to 27.1 min, 100% B to 5% B, hold for 12.9 min. The HILIC gradient was 0 to 5.0 min, 95% B to 35% B, hold for 1.0 min; 6.0 min to 6.1 min, 35% B to 5% B, hold for 16.9 min; 23.0 min to 23.1 min, 5% B to 95% B, hold for 16.9 min. The flow rate was 0.4 mL/min for RPC and 0.3 mL/min for HILIC. The column temperature was 40 °C for both columns. The injection volume was 2 µL.

To avoid systemic bias, the samples were analyzed by 2DLC-MS in random order. All samples were first analyzed by 2DLC-MS in positive mode followed by 2DLC-MS in negative mode to obtain the full MS data of each metabolite. For quality control purposes, a pooled sample was prepared by mixing a small portion of the supernatant from each sample which was then analyzed by 2DLC-MS after injection of every six biological samples. The pooled sample was also analyzed by 2DLC-MS/MS in positive mode and negative mode to acquire MS/MS spectra for metabolite identification 23-25.

For 2DLC-MS data analysis, MetSign software was used for spectrum deconvolution, metabolite identification, cross-sample peak list alignment, normalization, and statistical analysis. To identify metabolites, the 2DLC-MS/MS data of pooled sample were first matched to our in-house MS/MS database that contained the parent ion m/z, MS/MS spectra, and retention time of 187 metabolite standards. The thresholds used for metabolite identification were MS/MS spectral similarity ≥ 0.4, retention time difference ≤ 0.15 min, and m/z variation ≤ 4 ppm. The 2DLC-MS/MS data without a match in the in-house database were then analyzed using Compound Discoverer software (Thermo Fisher Scientific, Inc., Germany), where the threshold of MS/MS spectra similarity score was set as ≥ 40 with a maximum score of 100. The remaining peaks that did not have a match were then matched to the metabolites in our in-house MS database using the parent ion m/z and retention time. The thresholds for assignment using the parent ion m/z and retention time were ≤ 4 ppm and ≤ 0.15 min, respectively.

### Glucose uptake assay

Glucose Uptake-Glo^TM^ Assay from Promega (J1341) was performed in accordance with the manufacturer's instructions. Briefly, 2 × 10^4^ cells (hepatocyte cell lines) were seeded in complete medium into a 96-well tissue culture plate. When cells achieved 50-60% confluency, HFD-Exo plus nanoparticles (1 × 10^6^) packed scramble or miR-375 and PBS only as control were added and incubated for 16 h at 37 °C in a CO_2_ incubator. Cells were treated with 1 nM of insulin for an additional 1 h. Medium was removed and cells were washed twice with PBS. 50 μl of 2-deoxyglucose (DG, 1 mM per well) was added and incubated for 1 h at room temperature. 25 μl of stop buffer was added and mixed briefly, and then 25 μl of neutralization buffer was added and the plate shaken briefly. 100 μl of 2DG6P detection reagent was added and the mixture shaken for an additional 3 h at room temperature. Luminescence was recorded with 135 gain efficiency using a SYNERGY H1 (BioTek) luminometer.

### Statistical analysis

Unless otherwise indicated, GraphPad Prism 7.0 (GraphPad software) were used for data analysis. The data are presented as values with standard deviation (mean ± SD). The significance of differences in mean values between two groups was analyzed using the Student's *t*-test (one tailed). In cases of more than two groups, differences between individual groups were analyzed via one-way (Bonferroni multiple comparison) or two-way ANOVA. Pearson correlation coefficient test was used for two variables such as miR-375 & indole. Differences were considered significant when the *P*-value was less than 0.05. *P* values >0.05 were considered not significant (NS). * < 0.05, ** < 0.01, *** < 0.001, ****<0.0001.

## Result

### miR-375-3p inhibits the expression of the aryl hydrocarbon receptor in a GDNP dose dependent manner

Administration of ginger extract can prevent HFD-induced obesity 26 and fructose overconsumption-induced insulin resistance in rats 27. However, the molecular mechanism in the cell underlying ginger extract mediated prevention of insulin resistance is still elusive. Ginger extract contains a number of nanoparticles, predominately of two types: including exosome-like nanoparticles (the pellet after 100,000g for 1 h centrifugation) which have a role in the protection of alcoholic induced liver damage and another type of ginger nanoparticle (the pellet after 10,000g for 1 h centrifugation) that is the most enriched of the two (5 × 10^12^ particles/g of ginger tissue). We referred to the latter one as GDNP in this study. Major features of the GDNP are provided in Figure S1A-C and include EM morphology and size.

**Figure 1:**
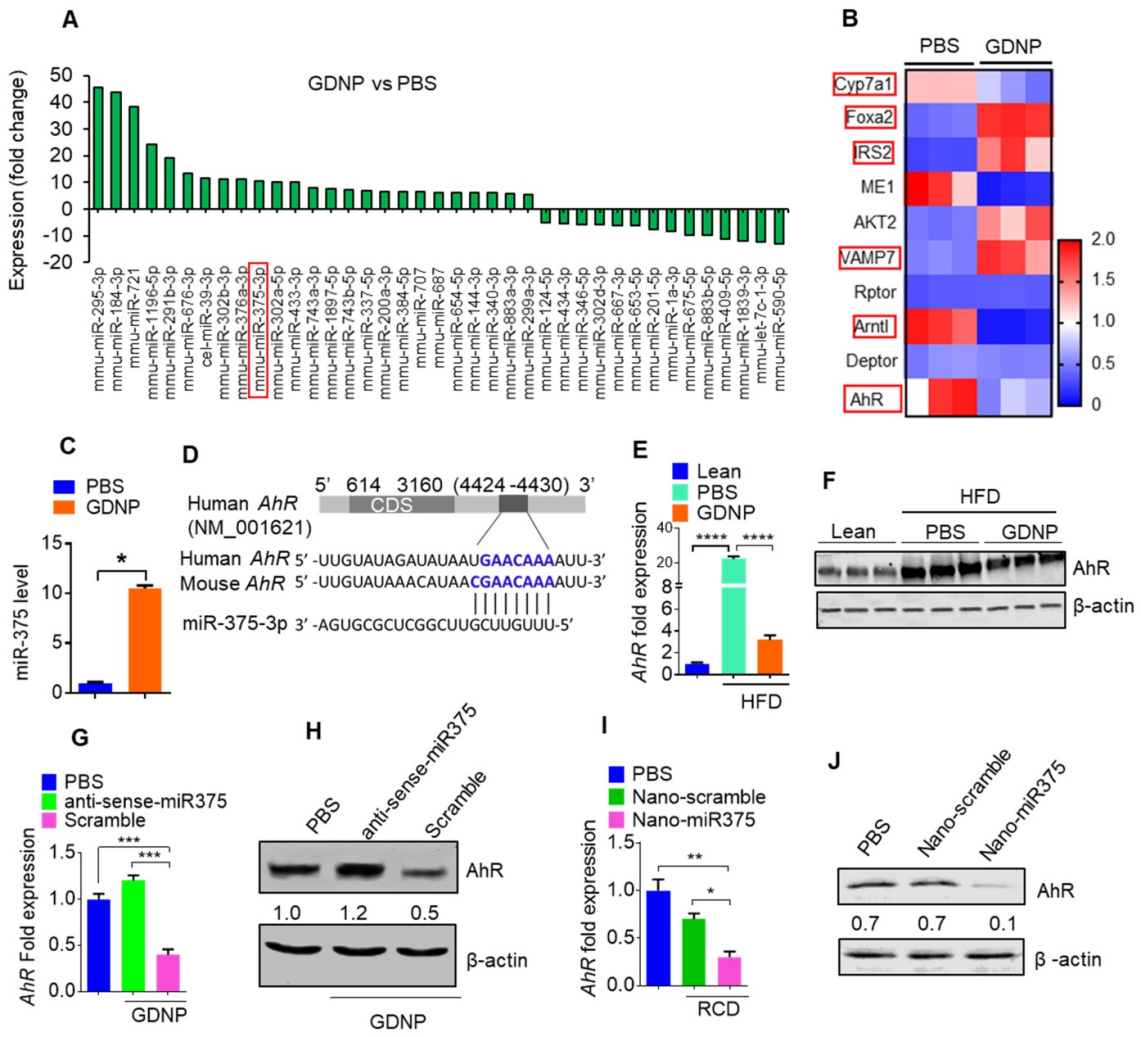
**miR-375 regulates AhR expression. (A)** Bar graph showing the fold-change in the expression of microRNAs in HFD mice small intestinal tissue induced by GDNP treatment, (expression following treatment with PBS served as the baseline value). The bar graph shows only microRNAs with >5 fold up- or downregulation. **(B)** Alteration of gene expression of small intestine (SI) tissues from high-fat diet (HFD)-fed mice treated with either PBS or GDNP (using an Affymetrix array). Red boxes highlighted are the genes involved in insulin signaling and lipid metabolism. **(C)** qPCR assay for miR-375 in cells cultured with PBS or GDNP for 12 h. **(D)** Graphical representation of the miR-375 binding site in AhR mRNA as predicted by TargetScan. **(E)** qPCR quantification of *AhR* expression in small intestine tissues of HFD-fed mice treated with PBS or GDNP. **(F)** Western blot presenting AhR expression in the small intestine tissues of lean and HFD-fed mice treated with PBS or GDNP. **(G & H)** *AhR* mRNA by qPCR (G) and protein expression by western blot (H) in MC-38 cells treated with PBS or GDNP with anti-sense-miR375 or scramble. Ratio to β-actin were shown (numbers in the middle of each panel).** (I & J)** *AhR* mRNA (I) and protein expression (J) in the small intestine tissues of RCD mice orally administered nanoparticles (Nano-scramble or Nano-miR375). Ratio to β-actin is shown in the middle as numbers. One-way ANOVA with the Bonferroni correction for multiple comparisons and the Student *t* test (one tailed) were used to calculate statistical significance (*p* value *<0.05; **<0.01; ***<0.001; ****<0.0001).

**Figure 2:**
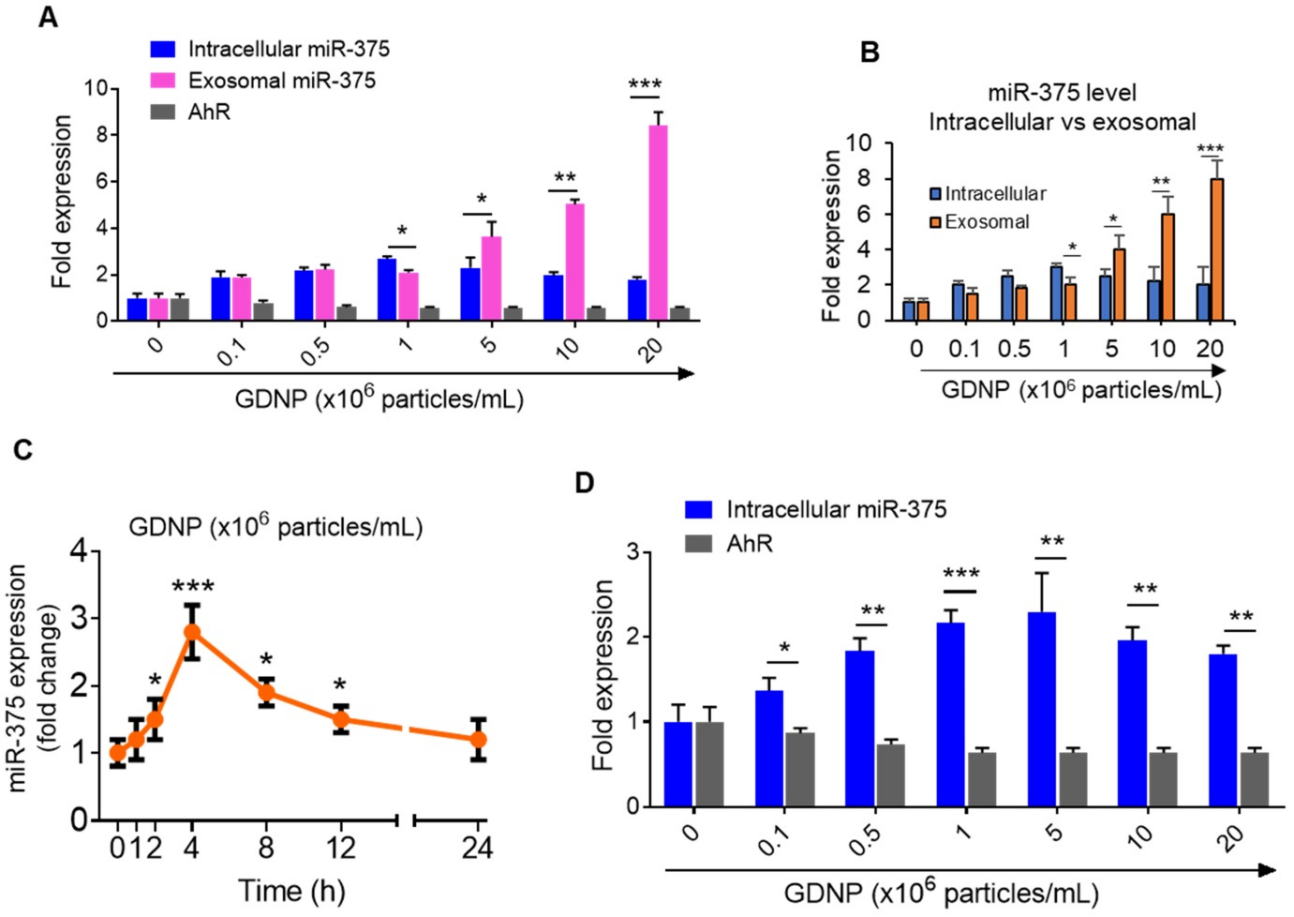
** GDNP induces miR-375 in MC-38 cells and sorts into exosomes. (A)** qRT-PCR for intracellular and exosomal miR-375 levels and AhR expression in MC-38 cells cultured with various concentrations of GDNP for 12 h. **(B)** qRT-PCR comparison for intracellular vs exosomal levels of miR-375 in MC-38 cells cultured with various concentrations of GDNP. **(C)** miR-375 levels expression in MC-38 cells cultured with GDNP in a time dependent manner. **(D)** miR-375 levels expression in FL83B (hepatocyte) cells cultured with GDNP in a dose dependent manner. One-way ANOVA with the Bonferroni correction for multiple comparisons and the Student *t* test (one tailed) were used to calculate statistical significance (*p* value *<0.05; **<0.01; ***<0.001).

The results generated from miRNA PCR microarray analysis of exosomes isolated from HFD fed mice gavage-given GDNP or PBS as a control indicate that GDNP treatment causes an alteration in the composition of exosomes (Figure 1A). miR-375 is one of these miRs changed due to GDNP treatment. Based on published literature, miR-375 plays a role in regulating multiple pathways including insulin signaling and lipid metabolism, and has important roles in the development of obesity and T2D 28. Genetic deletion of miR-375 results in a severely diabetic state 15. Therefore, we further investigated whether increased exosomal miR-375 plays a role in regulating insulin response in this study. Our cumulative findings suggested that GDNP is taken up by intestinal epithelial cells (Figure S2A-B) and GDNP treatment of HFD mice induced miR-375 expression (Figure 1A) and inhibited the expression of a number of genes (Figure 1B) in small intestinal tissues from GDNP-treated HFD mice compared to those treated with PBS. The *in vivo* data was reproduced in the *in vitro* cell cultures. A microRNA array confirmed by qPCR of wild-type (WT) MC-38 cells showed a more than 10-fold increase in the expression of miR-375 with GDNP treatment compared to PBS treatment (Figure 1C). With these findings in mind, we next explored the potential connection between the induction of miR-375 expression and the downregulation of genes listed in Figure 1B. To do so, we used TargetScan (http://www.targetscan.org) to predict the putative binding site in targeted genes. TargetScan results suggested that miR-375 has a strong potential target site at the 3' untranslated region (UTR) of *AhR* mRNA (Figure 1D; Figure S2C). Array and qPCR analyses of small intestinal tissue suggested that GDNP treatment of HFD mice resulted in a ~7-fold reduction in *AhR* mRNA levels compared to PBS treatment (Figure 1E), and western blotting revealed a reduction in AhR protein levels (Figure 1F) as well. Accordingly, GDNP treatment of MC-38 cells transfected with antisense-miR375 did not show downregulation of AhR at mRNA and protein levels (Figure 1G-H), suggesting that miR-375 induced by GDNP play a causative role in downregulating AhR. Next, we determined whether miR-375 regulates AhR *in vivo*. To this end, WT C57BL/6 RCD mice were administered orally daily for 14 days PBS or nanoparticles made from total lipids extracted from GDNP and packaged with miR-375 (nano-miR375, 20 nM) or scrambled RNA (Nano-scramble, 20 nM), and the resulting effects on AhR expression were assessed. qPCR and western blot analyses suggested a significant reduction in AhR expression in the small intestine of mice treated with Nano-miR375 compared to those treated with PBS or Nano-scramble (Figure 1I-J). However, adoptive transfer of nanoparticles did not affect the body weight of recipient mice (Figure S2D). Furthermore, we investigated whether *AhR* expression was reduced in a GDNP concentration-dependent manner in MC-38 cells. qPCR assays showed that the greatest reduction in *AhR* mRNA expression was observed at a concentration of 1 × 10^6^ GDNP/mL (Figure 2A; gray bars), and intracellular miR-375 expression was found to be the highest at the same concentration of GDNP (Figure 2A; blue bars). However, at concentrations higher than 1 × 10^6^ GDNP/mL, no further reduction in *AhR* mRNA expression was observed in GDNP treated MC-38 cells, and the levels of intracellular miR-375 began to decrease, whereas levels of exosomal miR-375 began to increase (Figure 2A; pink bars). Collectively, these results suggest that GDNP treatment induces the expression of miR-375. The increased expression of miR-375 inhibits *AhR* expression.

Interestingly, the intracellular expression of miR-375 peaked at a concentration of 10^6^ GDNP/mL and subsequently decreased with increasing concentrations of GDNP (Figure 2B). One possibility causing the reduction of intracellular miRNAs could be due to sorting of intracellular miRNAs into exosomes for intercellular communication 17. Exosomes were thus harvested from MC-38 cells treated for 12 h with various concentrations of GDNP and analyzed by qPCR. We found that the miR-375 level in exosomes increased with increasing concentrations of GDNP. Collectively, these data indicate that GDNP-stimulated upregulation of miR-375 leads to the sorting of miR-375 into exosomes in a GDNP dose- and time-dependent manner (Figure 2C). The reduction of AhR mRNA expression was also evaluated in the FL83B cells treated with GDNP and similar results were observed (Figure 2D).

### miR-375 is sorted into intestinal epithelial cell exosomes by the GDNP mediated induction of VAMP7

**Figure 3:**
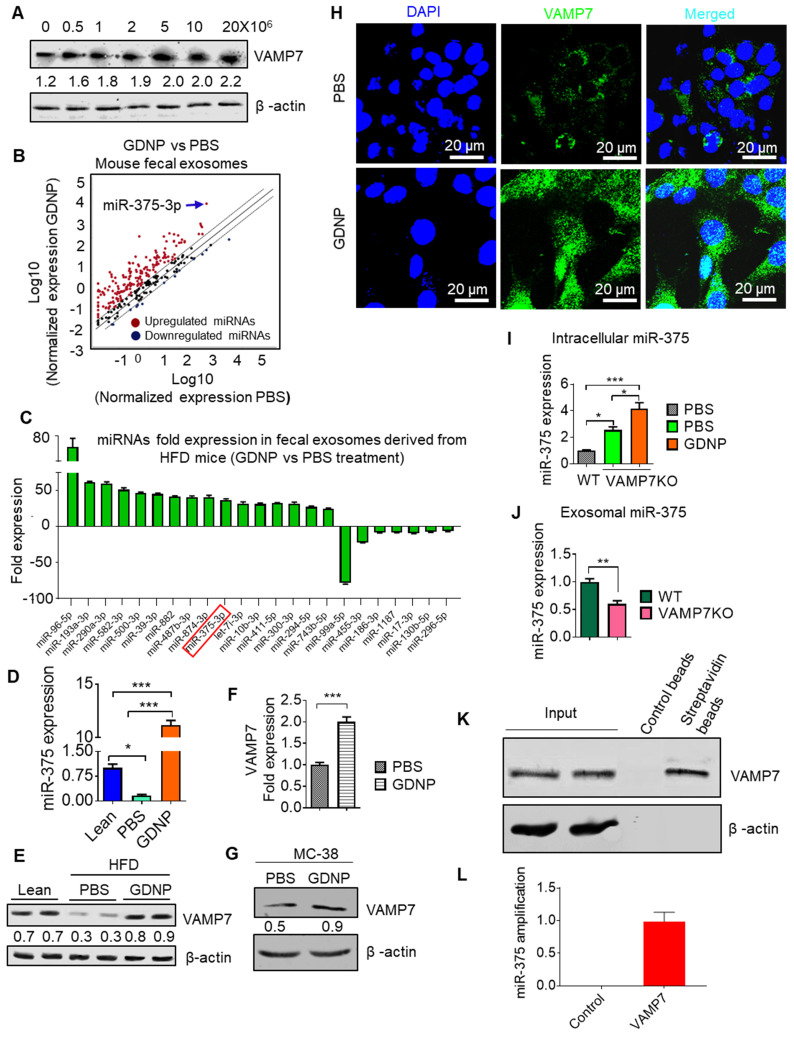
** miR-375 is sorted into exosomes via VAMP7 and intracellular miR-375 regulates AhR expression. (A)** Western blots for VAMP7 protein levels in MC-38 cells cultured with various concentrations of GDNP. Ratio of VAMP7 to β-actin is shown as a number between the two gels. **(B)** miRNA array expression profile of intestinal epithelial cell exosomes (A33^+^CD63^+^ exosomes) from feces derived from HFD-fed mice treated with GDNP vs PBS. **(C)** Bar graph showing HFD-fed mouse fecal exosomal miRNAs with a fold change (>25-fold or <5-fold) following treatment with GDNP vs PBS. **(D)** qRT PCR analysis of miR-375 expression in intestinal epithelial cell exosomes (A33^+^CD63^+^) from lean and HFD-fed mice treated with GDNP vs PBS. **(E)** Western blot showing VAMP7 expression in the small intestine of lean and HFD-fed mice treated with PBS or GDNP. Ratio of VAMP7 to β-actin is shown as a number between the two panels. **(F & G)** qRT-PCR analysis (F) and western blot (G) of VAMP7 expression in PBS- or GDNP-treated MC-38 cells. **(H)** Confocal images displaying VAMP7 expression in PBS- or GDNP-treated MC-38 cells. **(I)** qPCR analysis of the intracellular expression of miR-375 in WT and VAMP7KO MC-38 cells. **(J)** qPCR analysis of the exosomal levels of miR-375 in WT and VAMP7KO MC-38 cells. **(K)** MC-38 cells were transfected with biotinylated miR-375 and pulled-down with streptavidin beads. Western blots were carried out for VAMP7 using eluted extract from streptavidin beads. **(L)** qPCR for miR-375 in pulled-down product used for cDNA preparation. One-way ANOVA with the Bonferroni correction for multiple comparisons and the Student *t* (two-tailed) test were used to calculate statistical significance (*p* value *<0.05; **<0.01; ***<0.001).

Using an Affymetrix array for the small intestine, we found that GDNP-treated HFD-fed mice also showed increased VAMP7 expression in the small intestine relative to PBS treated mice (Table S1). The induction of VAMP7 led to increasing exosomal miR-375 when MC-38 cells were treated with GDNP at concentrations higher than 1 × 10^6^ GDNP/mL (Figure 3A). It has been suggested that VAMP7 is involved in the biogenesis of the exosomes 29. Our *in vitro* data suggested that GDNP treatment resulted in the sorting of miR-375 into exosomes and led us to investigate whether the composition of intestinal epithelial cell-derived exosomal miRNAs is regulated by diet. We thus analyzed the miRNA levels in fecal exosomes from HFD-fed mice, which had been fed with GDNP- or PBS for 12 months. Both exosomes from GDNP- and PBS-treated HFD-fed mice were positive for CD63, CD81, CD9 (exosomal markers) and A33 (intestinal epithelial cell marker) as assessed by western blot (Figure S3A). miRNA array and qPCR data (Figure 3B; Table S2) revealed that fecal exosomes from HFD mice treated with GDNP (>12 months) contained significantly higher levels of miR-375 compared to those of PBS-treated HFD mice (Figure 3C-D). Western blots of the intestinal tissue extracts from HFD-fed mice treated with PBS showed substantially reduced VAMP7 expression relative to lean mice, while HFD-fed mice treated with GDNP showed increased expression of VAMP7 compared to PBS HFD mice (Figure 3E). qPCR and western blot results demonstrated that GDNP treatment of WT MC-38 cells increased the expression of VAMP7 compared to treatment with PBS (Figure 3F-G). Consistent with the western blot results, confocal images of MC-38 cells treated with GDNP showed increased expression of VAMP7 compared to treatment with PBS (Figure 3H). Next, we generated VAMP7 knockout (VAMP7KO) MC-38 cells to assess the role of VAMP7 in miR-375 sorting. The qPCR analysis of miR-375 expression in VAMP7KO cells and exosomes suggested that miR-375 accumulated within the cells (Figure 3I) and in turn was reduced two-fold in the exosomes as the result of VAMP7 knockout (Figure 3J). To determine whether VAMP7 directly interacts with miR-375, MC-38 cells were transfected with biotinylated miR-375 and pulled down with streptavidin beads. Western blot analysis of the pulldown product against VAMP7 indicated that VAMP7 indeed binds to miR-375 (Figure 3K). PCR from pull-down product (used for cDNA preparation) further confirmed that VAMP7 did indeed interact with miR-375 (Figure 3L; Figure S3B). These results suggest that VAMP7 monitors intracellular levels of miR-375 to prevent an uncontrolled reduction of *AhR* by sorting miR-375 into exosomes. Accumulated results indicate that GDNP induced VAMP7 has a role in restoring AhR homeostasis expression disrupted by an HFD. Collectively, GDNP induces VAMP7 for maintaining homeostatic AhR expression by sorting miR-375 into exosomes. The level of reduction of AhR provides a feedback loop signal for VAMP7 initiation of sorting miR-375 out of exosomes.

### Exosomal miR-375 inhibits the expression of the *E. coli tryptophanase* (*tnaA*) gene and its indole production

**Figure 4:**
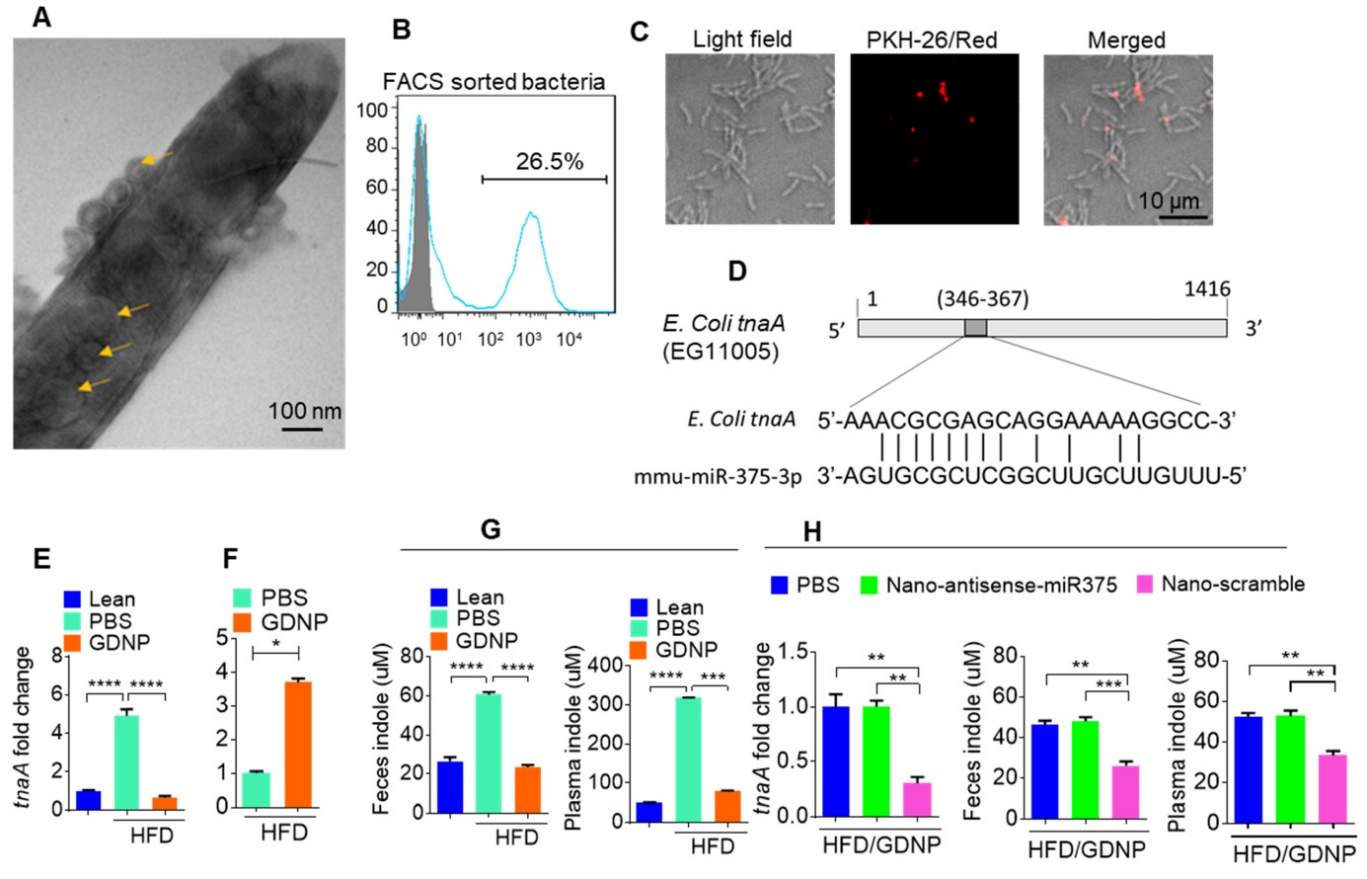
** Gut epithelial cell exosomes (CD63^+^A33^+^) influence gut bacterial populations and modulate microbial metabolites. (A)** Representative electron micrograph of gut bacteria containing fecal exosomes**.** Yellow arrows indicate exosomes inside and outside the bacteria. **(B)** FACS analysis of PKH26-positive gut bacteria from mice orally administered PKH-26-labeled fecal exosomes. **(C)** Confocal images of bacteria showing uptake of PKH-26-labeled fecal exosomes (red). **(D)** Schematic diagram of the putative targeting site of mmu-miR-375-3p in the *E. coli tryptophanase* (*tnaA*). **(E)** mRNA levels of the *tnaA* gene in gut bacteria derived from HFD mice treated with PBS or GDNP. **(F)** 2D LC-MS/MS analysis of unmetabolized tryptophan levels excreted into the feces of PBS- or GDNP-treated HFD-fed mice. **(G)** Quantification of the indole levels in the feces (h) and plasma (i) obtained from lean and HFD-fed mice that were treated with PBS or GDNP. **(H)** Fold change in* tnaA* gene expression in fecal bacteria (left), and indole estimation in the fecal supernatants (middle) and plasma (right) from GDNP-treated HFD-fed mice treated with PBS or nanoparticles packaged with antisense-miR375 or scramble. One-way ANOVA with the Tukey correction for multiple comparisons and the Student *t* (one tailed) test were used to calculate statistical significance (*p* value *<0.05; **<0.01; ***<0.001; ****<0.0001).

In the present study, treating mice with GDNP led to the induction of miR-375 expression and the sorting of this miRNA into intestinal epithelial exosomes by VAMP7. Intestinal epithelial cells (IECs) release miRNAs packed in exosomes that are released into the intestinal lumen. IEC exosomal miRNAs in turn influence the composition of gut bacterial populations 30. Using electron microscopy, we reveal that IEC-exosomes were taken up by gut bacteria (Figure 4A). To further confirm gut bacteria uptake of exosomes released by IECs, we orally administered PKH26-labeled epithelial cell-derived (CD63^+^A33^+^) fecal exosomes to wild-type C57BL/6 mice. We found that exosomes released into the lumen were indeed taken up by gut microbiota (Figure 4B-C). Analyses of PKH-26-positive FACS-sorted bacteria suggested that 26.5% of gut bacteria contained PKH-26 labeled fecal exosomes.

As miR-375 is increased in fecal exosomes from GDNP treated HFD-fed mice and intestinal exosomes are taken up by gut bacteria, we next hypothesized that exosomal miRNAs may regulate the expression of bacterial genes. To investigate this hypothesis, we performed a BLASTN search against the *E. coli* genome with the mmu-miR-375-3p sequence and found a putative target site for miR-375-3p in the *tryptophanase* (*tnaA*) gene of *E. coli* (Figure 4D). To determine whether GDNP-mediated induction of miR-375 affects the expression of *tnaA* mRNA*,* we harvested gut bacteria from HFD mice treated with PBS or GDNP and performed qPCR to assess the levels of the *E. coli tnaA* gene. We found that the gut bacteria from GDNP-treated HFD-fed mice showed approximately a five-fold decrease in *tnaA* gene expression compared to PBS-treated HFD-fed mice (Figure 4E). We reasoned that downregulation of the tryptophanase enzyme may affect tryptophan metabolism and, subsequently alter the levels of tryptophan-derived metabolites such as indole. We carried out 2D LC-MS/MS analysis of fecal metabolites and found that tryptophan was not completely metabolized in GDNP-treated HFD-fed mice, as evidenced by elevated levels of un-metabolized tryptophan excreted in their feces compared to PBS-treated mice (Figure 4F & Figure S4A). Moreover, the indole levels in the fecal supernatants and plasma from GDNP-treated HFD-fed mice were reduced (> 3- and 5-fold, respectively) compared to those in the PBS treated group (Figure 4G).

We further tested whether the reduction of indole levels in GDNP-treated HFD-fed mice is due to the increased expression of miR-375. GDNP lipid nanoparticles carrying an anti-sense miR-375 (Nano-antisense-miR375) or scrambled miR (Nano-scramble) were administered orally to GDNP-treated HFD-fed mice daily for 14 days. The qPCR results suggested that mice receiving Nano-antisense-miR375 had increased *tnaA* mRNA levels as well as an increase in the indole levels in the feces and plasma compared to nano-scramble treated mice (Figure 4H). However, nanoparticle adoptive transfer had no impact on body weight of recipient mice (Figure S4B).

### Human fecal exosomal miR-375 is negatively correlated with indole production

**Figure 5:**
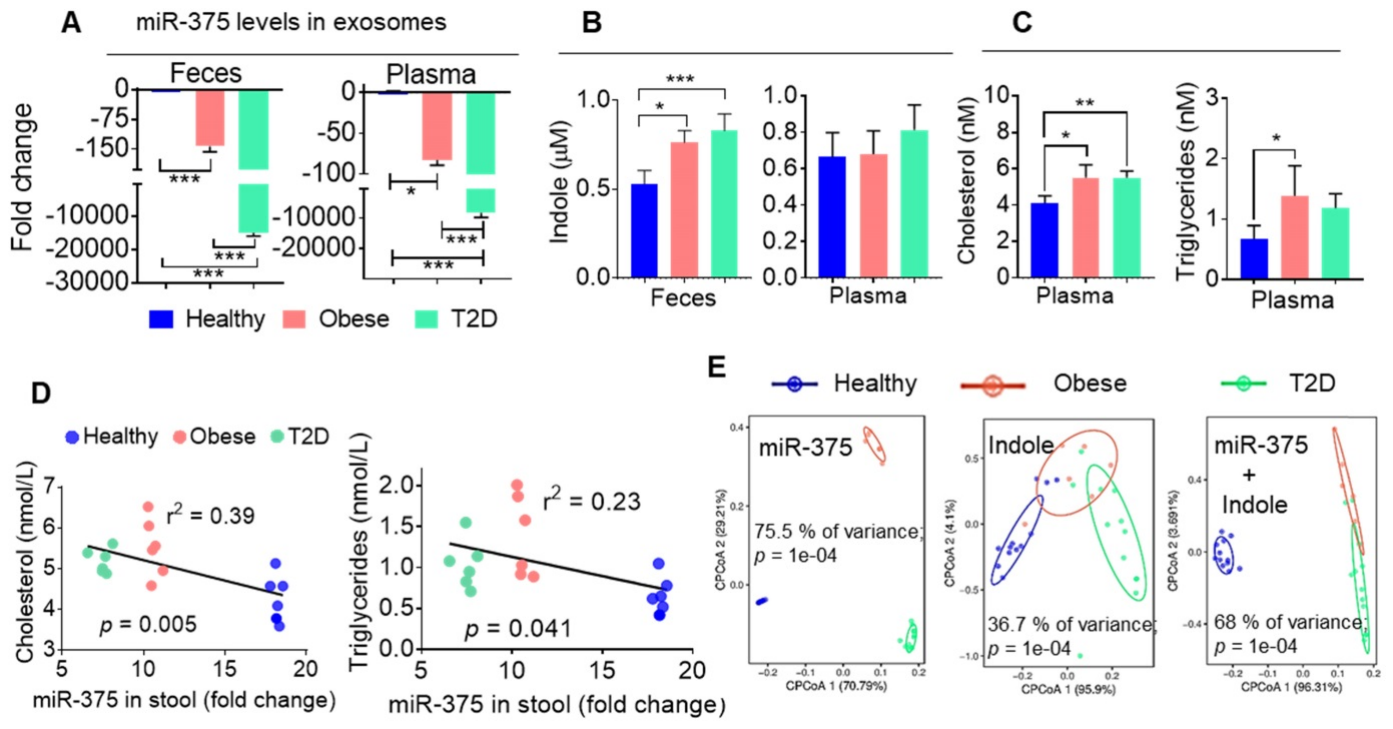
** Human fecal exosomal miR-375 is negatively correlated with indole production. (A)** qPCR analysis of miR-375 levels in human fecal exosomes and plasma exosomes derived from healthy, obese and individuals with T2D. **(B)** Quantification of indole levels in the feces and plasma. **(C)** Quantification of plasma cholesterol and triglyceride levels. **(D)** Scatter plot depicting the linear correlation between cholesterol and miR-375 levels, and triglycerides and miR-375 levels. **(E)** Principle component analysis (PCoA) of miR-375 and indole levels in obese, T2D and healthy human fecal exosomes. One-way ANOVA with the Bonferroni correction for multiple comparisons was used to calculate statistical significance. (*p* value *<0.05; **<0.01; ***<0.001).

To determine whether our findings generated from obese HFD-fed mice are applicable in patients with obesity-induced T2D, we isolated CD63^+^A33^+^ exosomes from the stool samples of healthy and obese individuals and patients with T2D. We then performed qPCR analysis of miR-375 levels in these exosomes. Consistently with our mouse data, we found that both obese patients and individuals with T2D showed large reductions in miR-375 expression in exosomes from feces and plasma (Figure 5A) compared to healthy controls. Furthermore, the indole levels in fecal supernatants (Figure 5B), but not in plasma, were increased in obese patients and patients with T2D compared to healthy individuals. Plasma cholesterol and triglyceride levels were also elevated in obese and T2D individuals relative to healthy controls (Figure 5C). Moreover, when linear correlation analysis was performed for miR-375 (feces exosomes) vs cholesterol and triglyceride levels, the clusters were highly segregated for healthy controls, obese patients and patients with T2D (Figure 5D). In addition to cholesterol and triglycerides, correlation analysis was performed for ALT, AST (Figure S5A) and adiponectin (Figure S5B) which showed segregation of the cluster according to the health status of individuals (healthy, obese and T2D). Principal component analysis (PCoA), which has been used to extract independent factors from inter-correlated factors, resulted in the classification of subjects into two groups; subjects with low fecal exosomal miR-375 and subjects with a high level of indole that is associated with obesity and the group with T2D. Collectively, the data supported the hypothesis that miR-375 expression is negatively correlated with fecal indole production (Figure 5E). These findings suggested that the level of miR-375 in fecal exosomes released by IECs is a critical factor for the inhibition of indole production and could be used as a prognostic biomarker/therapeutic agent for obesity induced T2D.

### miR-375 protects mice from fecal exosome-mediated insulin resistance transfer and glucose intolerance

**Figure 6:**
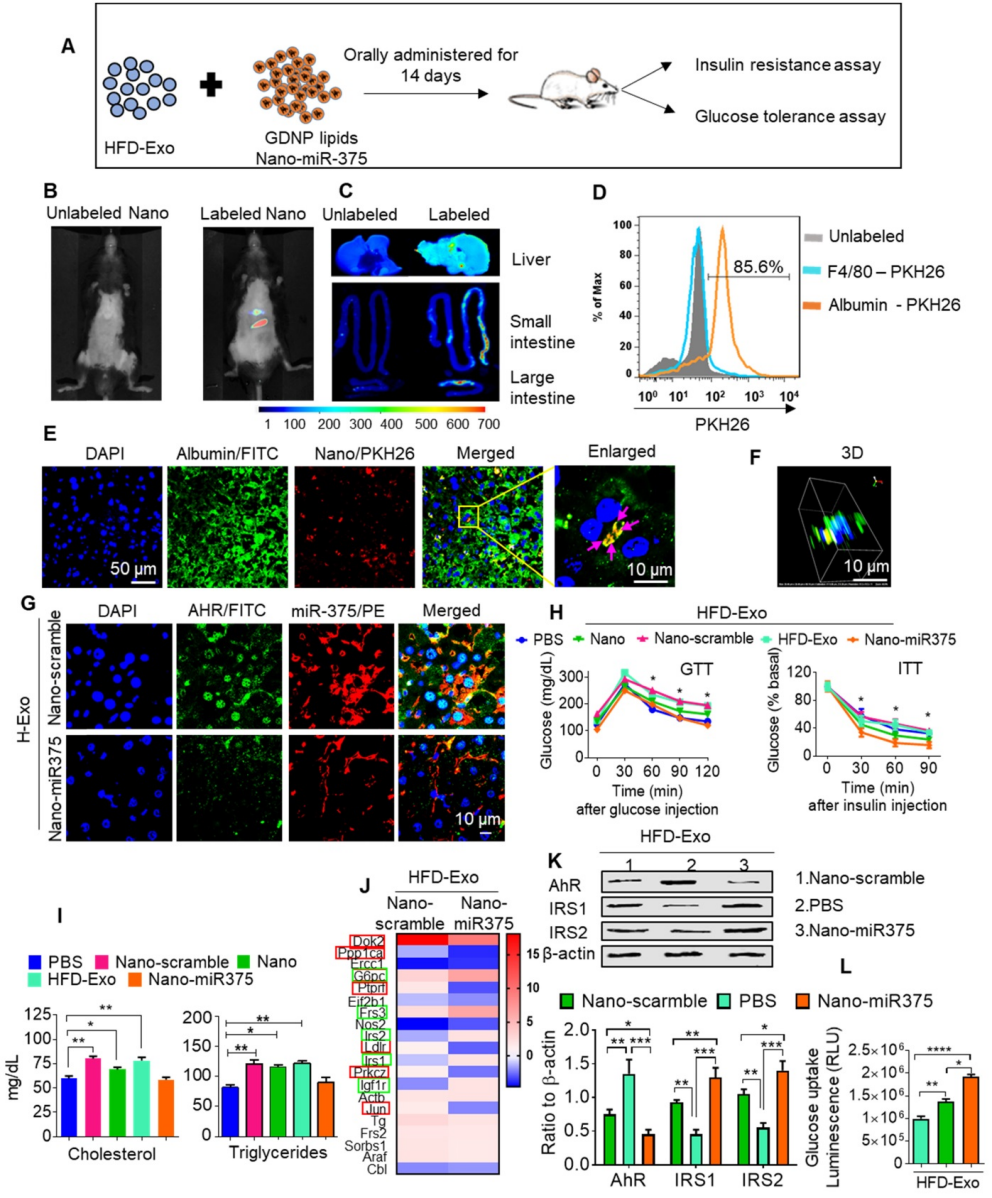
** miR-375 improves insulin sensitivity and glucose homeostasis and prevents dyslipidemia. (A)** Graphical representation of the experiment, which consisted of adoptive transfer of CD63^+^A33^+^ fecal exosomes (HFD-Exo) from HFD mouse (HFD fed 12 months) plus nanoparticles containing miR-375. Nanoparticles generated using the total lipid from GDNP. **(B)** Live imaging of mice orally administered PKH26 labeled nanoparticles containing miR-375. **(C)** Imaging of the liver, small and large intestines indicating the presence of labeled nanoparticles 6 hours after oral administration. **(D)** PKH26 labeled nanoparticle uptake by hepatocytes (albumin-positive cells) or Kupffer (F4/80-positive) cells. **(E)** Representative images of cellular uptake of PKH26-labeled nanoparticles by hepatocytes (albumin-positive cells). PKH26-labeled particles are indicated by pink arrows. **(F)** 3D image of PKH26-labeled nanoparticles in hepatocytes. **(G)** Confocal imaging to detect AhR (FITC) and biotinylated miR-375 or scrambled microRNA in liver tissues derived from mice orally administered nanoparticles. **(H)** GTT and ITT for C57BL/6 mice that received the fecal exosomes (HFD-Exo) along with nanoparticles containing miR-375 or scrambled RNA for 14 days while the mice were fed a HFD. Statistical comparisons were made between HFD-Exo vs Nano-miR375; HFD-Exo was responsible for insulin resistance and miR-375 responsible for preventing the development of insulin resistance. Nanoparticles contained scramble RNA (Nano-scramble); nanoparticles only (Nano); and nanoparticles contained miR-375 (Nano-miR375). **(I)** Cholesterol and triglyceride levels in plasma derived from HFD mice treated with either PBS or nanoparticles (above mentioned) for 14 days. **(J)** Insulin signaling array of mouse hepatocytes cultured with fecal exosomes (HFD-Exo) along with nanoparticles (contained scramble & nano-miR-375) showing alterations in genes involved in insulin signaling. Green-boxed genes promote insulin activity and red-boxed genes inhibit insulin activity. **(K)** Western blot depicting Foxa2, AhR, and IRS-1 and 2 expression in hepatocytes treated with fecal exosomes (HFD-Exo) along with nanoparticles (contained scramble & nano-miR-375). The ratio to β-actin is shown in bar graph form as part of panel K. **(L)** Effect of fecal exosomes (HFD-Exo) on glucose uptake by hepatocytes. One-way ANOVA with a Bonferroni correction for multiple comparisons test was used to calculate statistical significance. (*p* value *<0.05; **<0.01; ***<0.001; ****<0.0001).

*In vivo* administration of fecal exosomes from HFD-fed mice induced insulin resistance and glucose intolerance 31. Here, we also showed that intestinal exosomes from HFD-fed mice (HFD-Exo) can traffic to the liver and subsequently be taken up by hepatocytes (major features including liver cells taking up HFD-Exo and development of insulin resistance are included in Figure S6A-F). Based on these data, we then hypothesized that GDNP treatment leads to altering the composition of exosomes, and increased exosomal miR-375 plays a predominant role in reversing HFD-Exo mediated development of hepatocyte insulin resistance by decreasing AhR-mediated signaling in hepatocytes. To investigate this hypothesis, we packaged miR-375 (Nano-miR375) or scrambled RNA (Nano-scramble) into ginger nano-vector and orally administered it daily for 14 days along with fecal exosomes (CD63^+^A33^+^, HFD-Exo) isolated from B6 mice fed the HFD for 12-months to lean C57BL/6 mice (Figure 6A). Live imaging of the mice suggested that PKH26 and DIR dye double-labeled ginger nano-vectors were transported to the liver within 6 h post-administration (Figure 6B-C). Further analysis of cellular uptake was explored by flow cytometry and confocal microscopy. We found that ginger nano-vectors were taken up by hepatocytes (albumin^+^) (Figure 6D-F). Functional analyses showed that the group that received HFD-Exo plus Nano-miR375 had lower levels of AhR (Figure 6G) and improved glucose tolerance and insulin sensitivity compared to all other groups including the nano-scramble group, nano-vector group, and PBS group (Figure 6H). However, mice that received only HFD-Exo developed glucose intolerance and insulin resistance. Further, the role of miR-375 mediated downregulation of AhR expression in insulin response was demonstrated. AhR knockout (AhRKO) mice and C57BL/6 mice were orally administered HFD-Exo for 14 days while being fed the HFD. AhRKO mice exhibited no glucose intolerance or insulin resistance compared to the C57BL/6 mice (Figure S7A-B respectively).

Insulin resistance can also alter systemic lipid metabolism, which then leads to the development of dyslipidemia. Nano-miR375 treatment inhibited the development of dyslipidemia, as evidenced by restoring homeostasis of blood cholesterol and triglycerides (Figure 6I). Next the Nano-miR375 treatment effects on expression of hepatic genes that regulates insulin signaling were assessed using an insulin signaling PCR array. After 24 h in culture, primary mouse hepatocytes were co-cultured with HFD-Exo (100 ng/mL) plus Nano-miR375, Nano-Scramble-miR (20 nM) or PBS as a control for an additional 24 h. RNA was extracted from 24 h treated hepatocytes and used in the insulin signaling PCR array (Qiagen). The array data suggested that the expression of *G6Pc*, *Frs3*, *IRS-1*, *IRS-2*, and *IGF1R* were upregulated (Figure 6J, green boxes), whereas *DoK2*, *Ppp1ca*, *Ptprf*, *Ldlr*, *PrkcZ*, and Jun (Figure 6J, red boxes) were downregulated after Nano-miR375 treatment compared with hepatocytes treated with Nano-Scramble. Protein levels were confirmed by western blot analyses (Figure 6K). The PBS plus HFD-Exo treated hepatocytes also showed inhibited glucose uptake (Figure 6L). However, these inhibitory effects were dampened in GDNP exosome-treated hepatocytes. Moreover, plasma levels of free amino acids associated with T2D 32 were elevated in HFD-fed mice, whereas GDNP treatment reduced plasma free amino acids (Figure S8A). Free amino acids which have been known to be beneficial in T2D were upregulated in GDNP-treated HFD mice (Figure S8B). Collectively, these data suggest that oral delivery of miR-375 packed in GDNP-based nanovectors prevent glucose intolerance and insulin resistance induced by fecal exosomes of HFD-fed mice.

The role of miR-375 in increasing insulin response to glucose uptake was further demonstrated in FL83B cells treated with fecal exosomes isolated from HFD mice gavage-given GDNP along with nanoparticles packaged with antisense-miR375 or scrambled miR. The cells treated with antisense-miR375 exhibited lower levels of glucose uptake compared to scramble miR treated cells (Figure S8C), suggesting that fecal exosomal miR-375 plays a critical role in enhancing insulin response.

## Discussion

A HFD is known to change cellular physiology and lead to the development of detrimental health outcomes such as obesity or T2D. Studies on mice and humans have suggested that chronic consumption of a HFD causes over-expression of AhR. Studies have further suggested that AhR overexpression inhibits the insulin response 33. However, it is unclear whether diet-derived factors modulate the expression of AhR.

Here, we demonstrated that ginger-derived nanoparticles (GDNP) can prevent insulin resistance by restoring homeostasis in gut epithelial AhR signaling in mice fed a HFD. GDNP inhibits AhR expression by induction of miR-375. GDNP mediated induction of miR-375 inhibits AhR expression via a potential binding site in the AhR 3' UTR. This process occurs in a tightly regulated manner, as evidenced by the fact that GDNP treatment not only induced the expression of miR-375 but also of VAMP7, which monitors intracellular levels of miR-375. When intestinal epithelial cells received a GDNP dose higher than 1 × 10^6^ GDNP/mL, miR-375 was sorted into exosomes in a VAMP7-dependent manner to reduce the intracellular level of miR-375 and prevent further reduction of AhR. These findings provide a foundation for future studies to determine the cellular machinery that monitors the level of intracellular miR-375 and AhR which underlie the timing for sorting of miR-375 into exosomes in a GDNP dose-dependent manner.

Intestinal epithelial miRNAs released into the lumen have been suggested to modulate the composition and function of gut microbiota 30. Our data show that exosomal miR-375 targets the bacterial *tnaA* gene and ultimately inhibits the production of indole, a known AhR ligand 34.

Our result show that a region other than the "seed sequence" of miR-375 is able to target the 3' end of tnaA and subsequently down-regulate tnaA. This result agreed with published literature, suggesting that non-seed sequence matches, or imperfect seed matches also plays a significant role in functionality 35-37. The 3' end base pairing of miRNAs or center region base pairing can provide the platform to mediate gene regulation 38-40. Even though these sites might be small in length, mutation studies have suggested that non-seed pairing also plays significant role in gene downregulation 41-43.

Moreover, GDNP treatment led to decreased indole production by increasing exosomal miR-375 - as a result, AhR over-activation was prevented and AhR homeostasis was maintained. Additionally, AhR has multiple ligands, including endogenous metabolites, nutrients and factors released by gut microbiota. Ligand-dependent activation of AhR can result in an extremely diverse spectrum of biological effects that occur in a ligand-, species- and tissue-specific manner.

In a healthy, varied diet, multiple particles of variable size and composition are ingested, each of which has a distinct effect on the regulation of AhR activity.

## Conclusion

In summary, our results demonstrate that ginger-derived nanoparticles (GDNP) prevent obesity and insulin resistance via induction of expression of miR-375 and VAMP7. Intracellular miR-375 inhibits high-fat diet induced AhR which contributes to insulin resistance. Sorting of miR-375 into exosomes via VAMP7 leads to prevention of insulin resistance mediated by bacterial indole and hepatic AhR. Our findings support further exploration of the development of edible nanoparticle-based strategies for the prevention and treatment of metabolic disease (see the Graphical abstract).

The finding that miR-375 can be delivered via nano-vectors made up of GDNP lipids to target the *E. coli tnaA* gene and the hepatocyte to regulate the expression of insulin response genes, suggest that the development of carriers to deliver miR-375 as an oral therapeutic to targeted tissues would represent a significant advance in the treatment of disease. In addition, this strategy would likely have few side effects because the system is based on edible plant carriers to deliver therapeutic agents to the gut bacteria and liver.

## Supplementary Material

Supplementary figures and tables.
