# Supplementary material for: miR-375 prevents high-fat diet-induced insulin resistance and obesity by targeting the aryl hydrocarbon receptor and bacterial tryptophanase (tnaA) gene
Source: Theranostics. 2021 Feb 19;11(9):4061–77. doi: 10.7150/thno.52558 (PMC7977461; doi:10.7150/thno.52558)
Supplement: Supplementary file 1 — Supplementary figures and tables. [file thnov11p4061s1.pdf]

Supplementary Material

miR-375 prevents high-fat diet-induced insulin resistance and obesity by targeting the aryl hydrocarbon receptor and bacterial tryptophanase (*tnaA*) gene

Anil Kumar[1#], Yi Ren[2#] Kumaran Sundaram[1], Jingyao Mu[1], Mukesh K Sriwastva[1], Gerald W Dryden[1,3], Chao Lei[1], Lifeng Zhang[1], Jun Yan[1], Xiang Zhang[4], Juw Won Park[5,6], Michael L Merchant[7], Yun Teng[1*], and Huang-Ge Zhang[1,8,9,*]

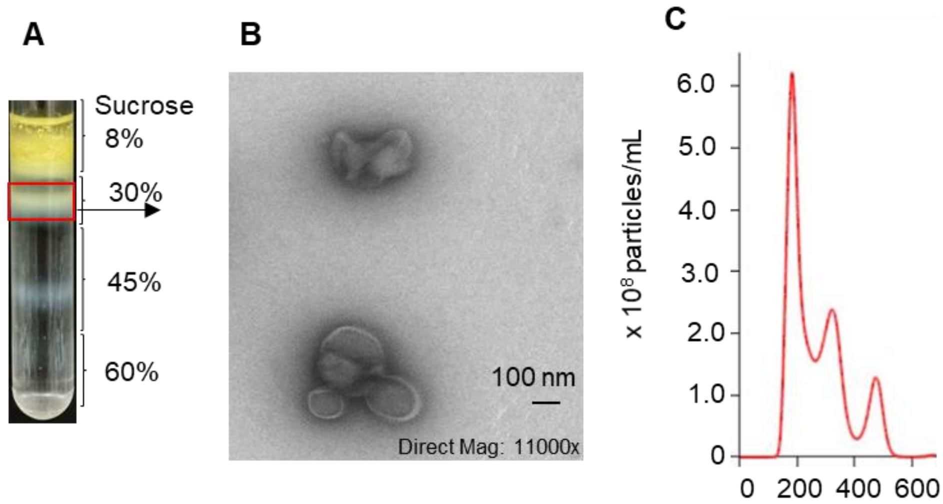

Figure S1. Characterization of ginger nanoparticles (GDNP).

- **A.** Depiction of the sucrose gradient purification of ginger-derived nanoparticles (GDNP).
- **B.** Electron micrograph of purified GDNP from the red box in panel A.
- **C.** GDNP size distribution, as determined using the Nano-sight NS300.

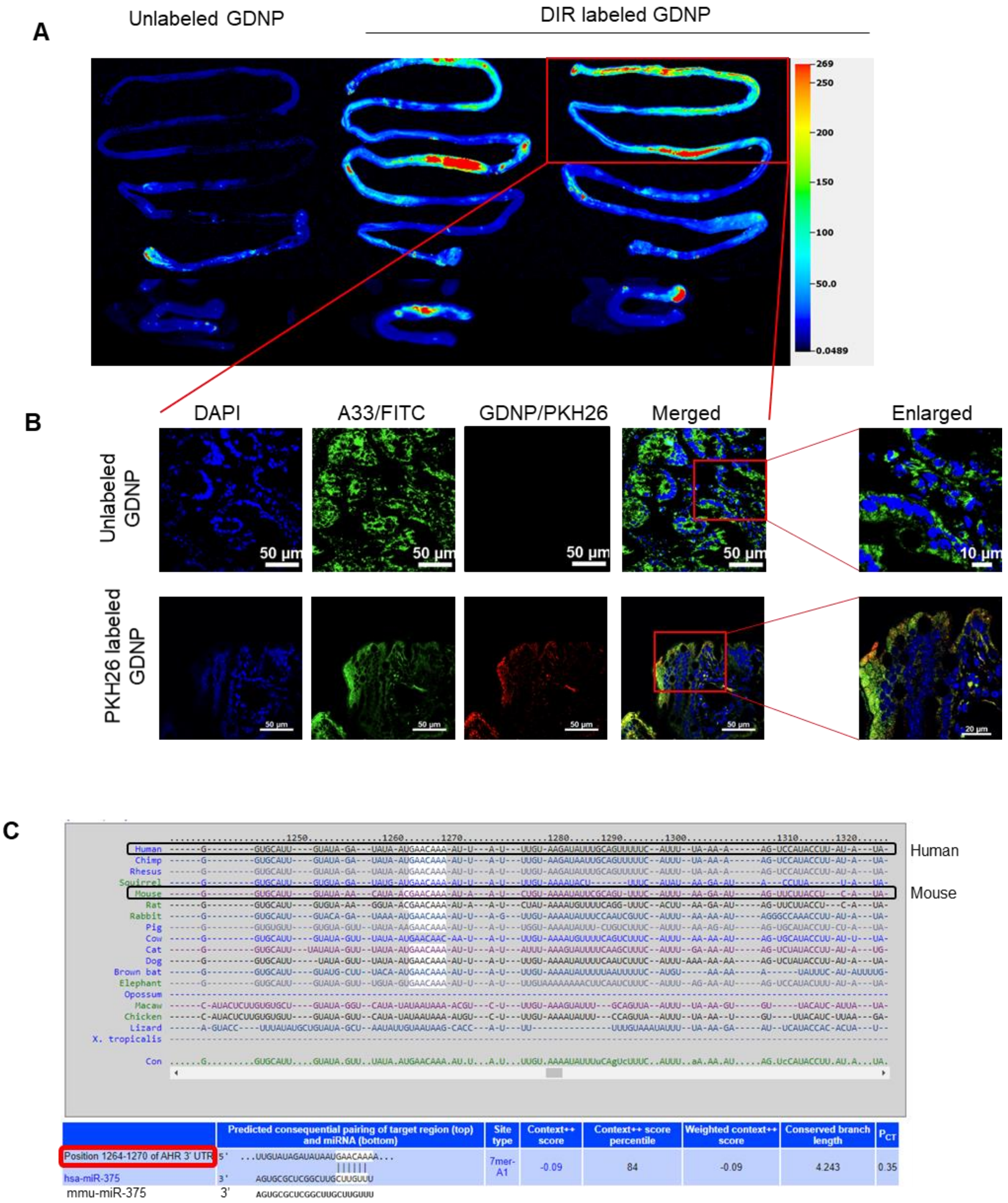
A
Unlabeled GDNP
DIR labeled GDNP
269
250
200
150
100
50.0
0.0489
B
DAPI
A33/FITC
GDNP/PKH26
Merged
Enlarged
Unlabeled GDNP
PKH26 labeled GDNP
50 μm
10 μm
20 μm
C
Human
Mouse
Predicted consequential pairing of target region (top) and miRNA (bottom)
Site type
Context++ score
Context++ score percentile
Weighted context++ score
Conserved branch length
Position 1264-1270 of AHR 3' UTR
hsa-miR-375
mmu-miR-375
7mer-A1
-0.09
84
-0.09
4.243
0.35

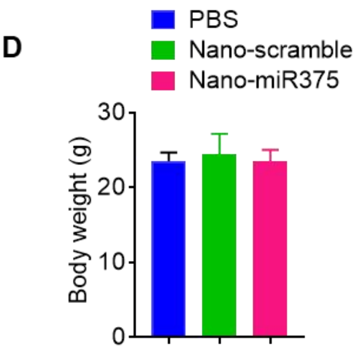
D
PBS
Nano-scramble
Nano-miR375
Body weight (g)
30
20
10
0

Figure S2. GDNP uptake by small intestine epithelial cells.

- **A.** Scanning image of intestine showed the signals from DIR labeled GDNP. The region of the intestine was used to prepare the section for confocal microscopy shown in panel B.
- **B.** GDNP uptake by small intestine epithelial cells. Scale bar as indicated.
- **C.** TargetScan screenshot depicting the sequence target of miR-375 in *AhR* mRNA 3'UTR. Black boxes showing sequence homology between human and mouse *AhR*.
- **D.** Body weight of mice receiving adoptive transfer of nanoparticles for 14 days.

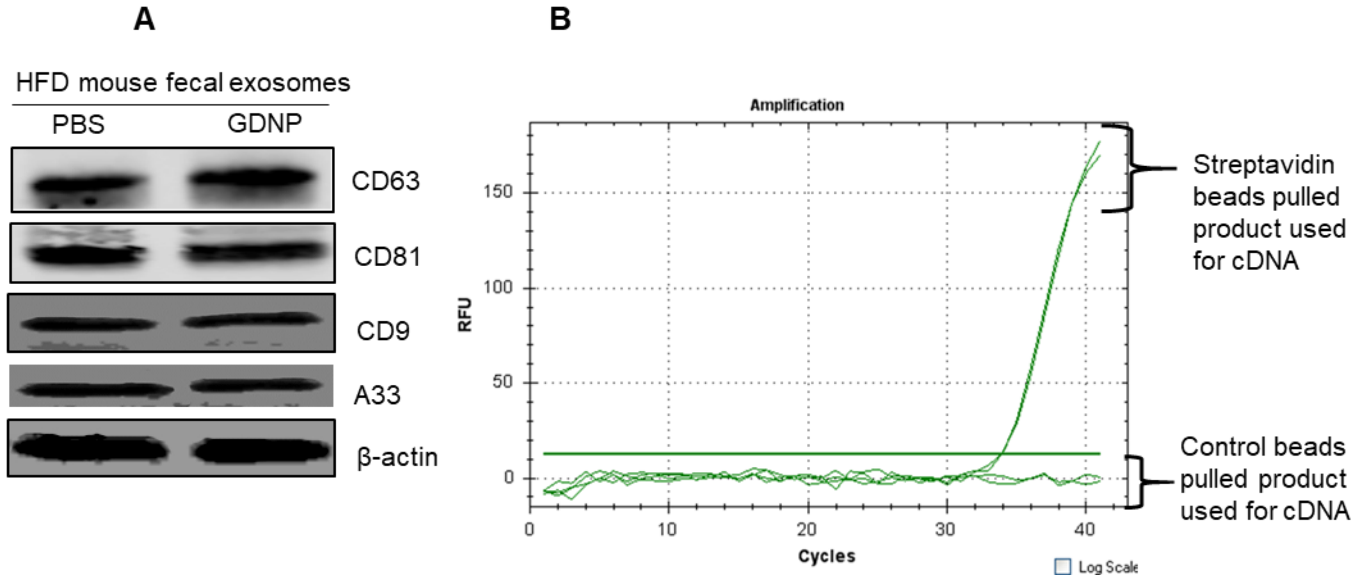

Figure S3. Characterization of Intestinal epithelial cells exosomes

- **A.** Western blot images showing the expression of CD63, CD81, CD9 (exosomal markers) and A33 (intestinal epithelial cell marker) on fecal exosomes of HFD mice treated with PBS or GDNP.
- **B.** qPCR for miR-375 in control and streptavidin bead pulled down products.

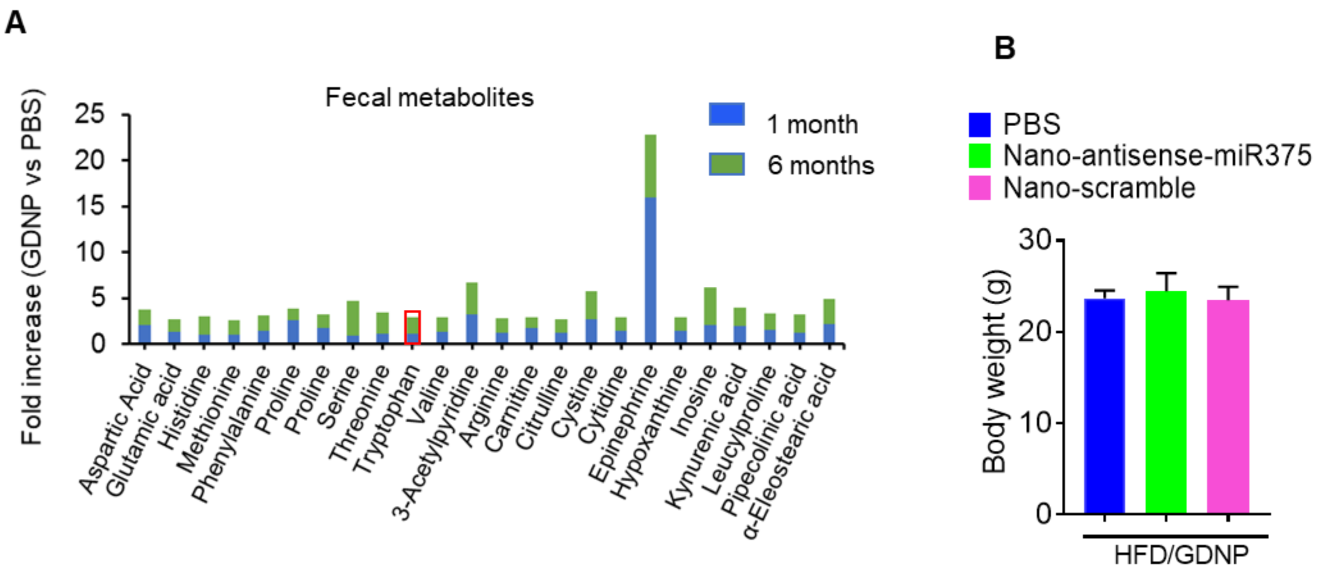

Figure S4. miR-375 regulates tryptophan metabolism and indole production

- **A.** A 2D LC-MS analysis of fecal supernatants of HFD-fed mice treated with PBS or GDNP for 1 month or 6 months.
- **B.** Body weight of mice received adoptive transfer of nanoparticles packaged with or Nano-antisense-miR375 or scrambled miRNA along with exosomes derived from GDNP HFD mice.

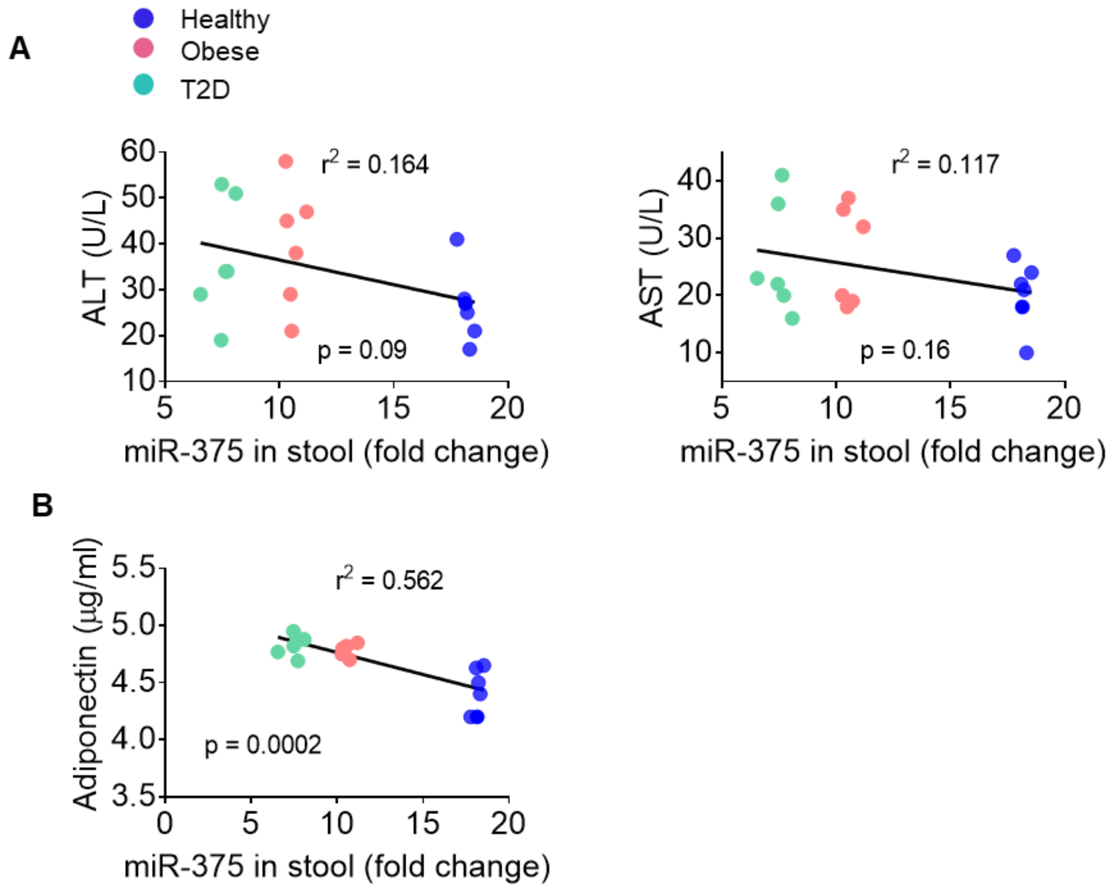

Figure S5. miR-375 correlated as biomarker with disease progression

- **A.** Scatter plot depicting the linear correlation between ALT vs miR-375 and AST vs miR-375 levels (human stool exosomes).
- **B.** Scatter plot depicting the linear correlation between adiponectin vs miR-375 levels (human stool exosomes).

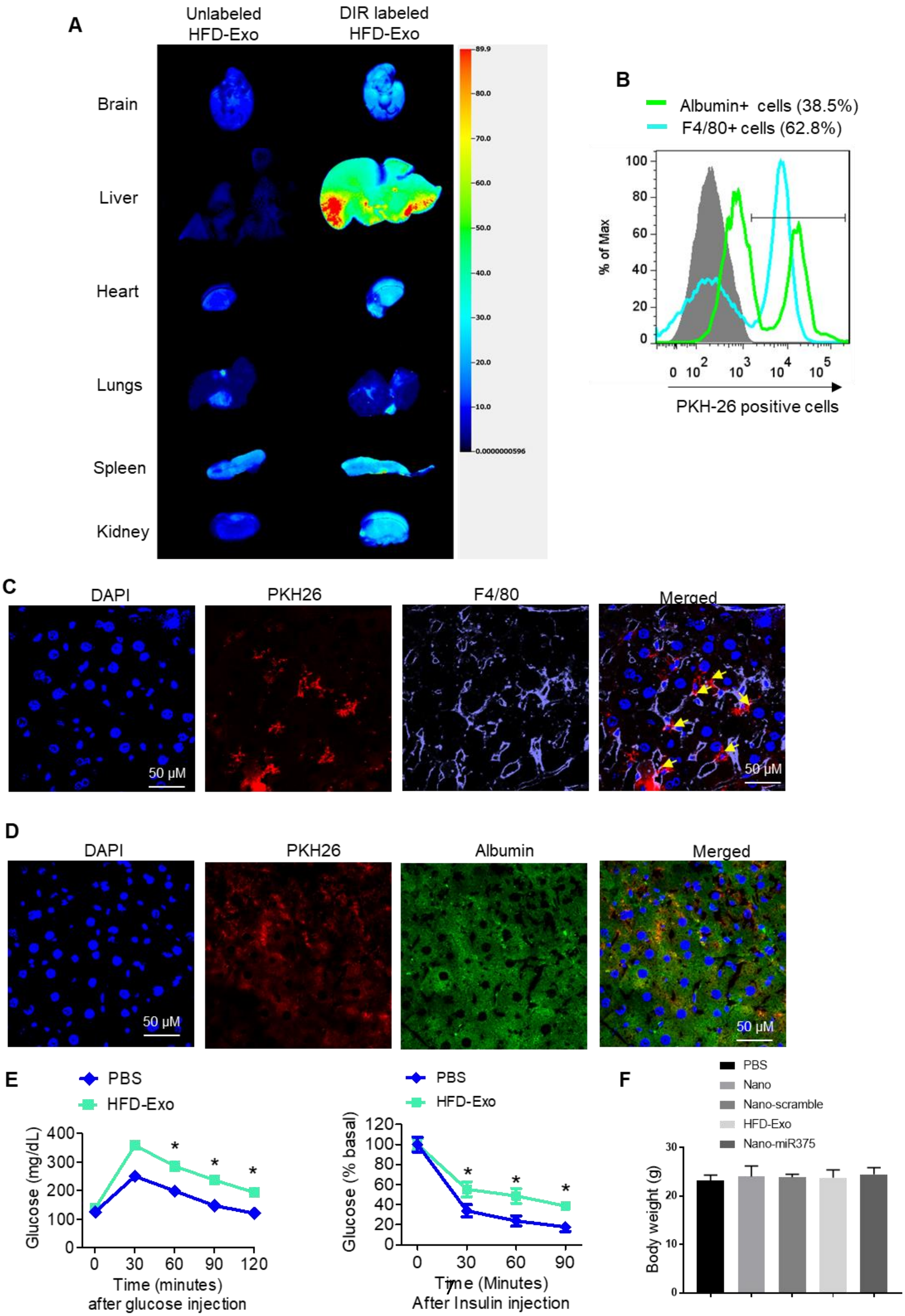
A
Unlabeled HFD-Exo
DIR labeled HFD-Exo
Brain
Liver
Heart
Lungs
Spleen
Kidney
89.9
80.0
70.0
60.0
50.0
40.0
30.0
20.0
10.0
0.0000000596
B
Albumin+ cells (38.5%)
F4/80+ cells (62.8%)
% of Max
PKH-26 positive cells
C
DAPI
PKH26
F4/80
Merged
50 μM
D
DAPI
PKH26
Albumin
Merged
50 μM
E
PBS
HFD-Exo
Glucose (mg/dL)
Time (minutes)
after glucose injection
Glucose (% basal)
Time (Minutes)
After Insulin injection
F
PBS
Nano
Nano-scramble
HFD-Exo
Nano-miR375
Body weight (g)

Figure S6. Uptake of HFD-Exo by liver F4/80 cells and developed the insulin resistance.

- **A.** Scanned organ images demonstrating preferential localization of HFD-Exo to the liver.
- **B.** Flow cytometry analysis of PKH-26-labeled exosome uptake by hepatocytes (Albumin^{+}) and Kupffer cells (F4/80^{+}). Percentage of cells summarized at right.
- **C. & D.** PKH26-labeled HFD exosomes visualized by confocal microscopy in Kupffer cells/F4/80/purple (yellow arrow; C) and hepatocytes/albumin^{+}/green (D).
- **E.** Glucose tolerance (upper) and insulin tolerance (lower) tests for mice receiving the HFD-EXo for 14 days while mice were fed HFD.
- **F.** Body weight of mice receiving adoptive transfer of HFD-Exo along with nanoparticles packaged with scramble or miR-375 for 14 days.

Student t test (two-tailed) was used to calculate statistical significance. (*p* value *<0.05).

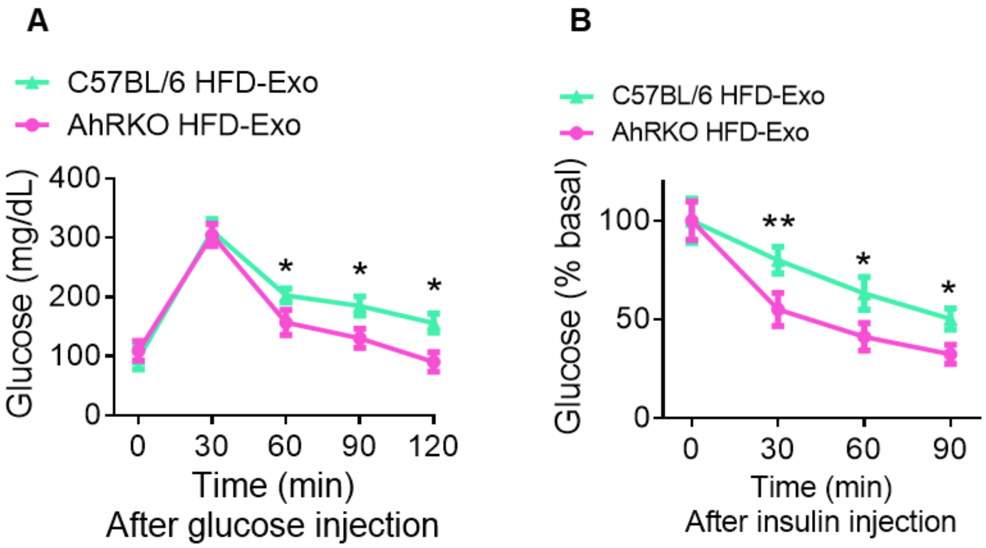

Figure S7. HFD-Exo induced insulin resistance via AhR.

- **A.** Glucose tolerance test of C57BL/6 and AhR KO mice receiving adoptive transfer of HFD-Exo for 14 days while fed HFD.
- **B.** Insulin tolerance test of C57BL/6 and AhR KO mice receiving adoptive transfer of HFD-Exo for 14 days while fed HFD.

Student t test (two-tailed) was used to calculate statistical significance. (*p* value *<0.05; **<0.01).

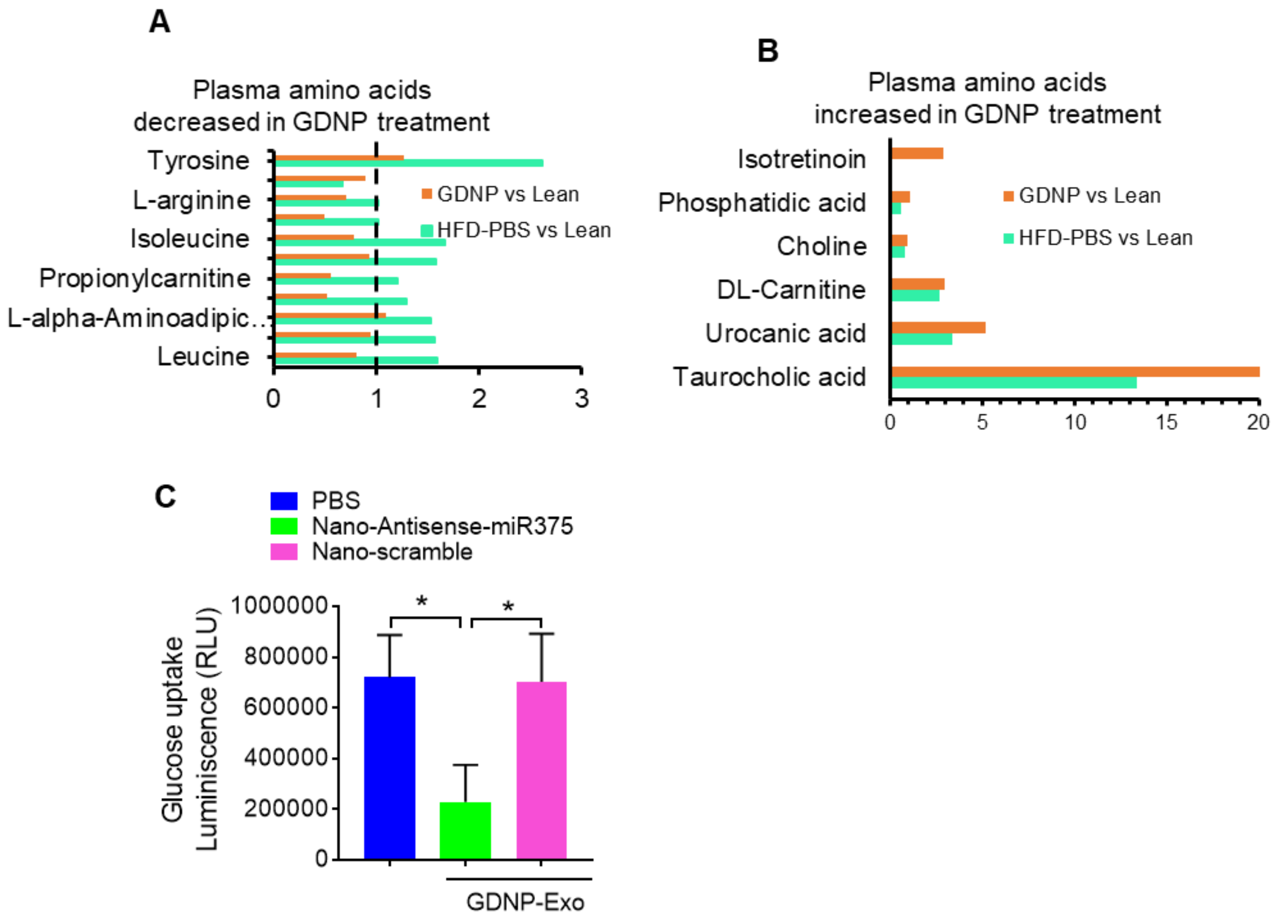

Figure S8. Plasma levels of free amino acid in HFD-fed mice treated with PBS or GDNP vs control lean mice

A. & B. 2DLC-MS mass spectrometry analysis was performed to analyze levels of plasma amino acids. Data are shown as fold change in measured levels as normalized to levels observed in lean control mice (dotted line) with decreases shown in (A) and increases in (B).

C. Glucose uptake by FL83B cells treated with fecal exosomes isolated from HFD mice gavage-given GDNP along with nanoparticles packaged with antisense-miR375 or scramble miRNA for 16 hours followed by 1nM insulin treatment for 1 hour prior to cells were harvested.

One-way ANOVA with the Tukey multiple comparison was used to calculate statistical significance. (*p* value *<0.05).

Table S1. Log2 values for the expression of genes in the Affymetrix array of small intestine tissues of HFD-fed mice treated with PBS or GDNP.

	PBS1	PBS2	PBS3	GDNP1	GDNP2	GDNP3
Vamp7	0.551879	0.568156	0.572387	0.674514	0.642258	0.595921
AhR	0.410544	0.402235	0.437675	0.386698	0.356065	0.339581
AKT1	0.659002	0.611732	0.569033	0.652737	0.643376	0.564498
FGF15	0.771172	0.66257	0.680268	0.843437	0.805478	0.76957
NR1H4	0.63152	0.707821	0.699273	0.781636	0.717719	0.70011
ARNTL	0.549635	0.577095	0.622694	0.530312	0.502515	0.496692
Cyp8b1	0.226584	0.257542	0.223589	0.247204	0.227501	0.23043
mTor	0.430174	0.47933	0.427613	0.393172	0.481274	0.46527
Rptor	0.319686	0.353631	0.350475	0.388464	0.374511	0.379824
Deptor	0.46046	0.575978	0.593069	0.532666	0.5109	0.573319
Cyp7a1	0.249019	0.265363	0.256568	0.247793	0.231414	0.235943
AKT2	0.421761	0.417318	0.424259	0.420247	0.409167	0.404079

Table S2. miRNA in fecal exosomes derived from HFD-fed mice treated with PBS or GDNP. Only microRNAs with ≥2-fold changes following treatment with GDNP vs PBS are listed. A negative fold change indicates downregulation.

Mature ID	Fold change	Mature ID	Fold change	Mature ID	Fold change
mmu-miR-96-5p	76.06	mmu-miR-19a-3p	20.52	mmu-miR-190b-5p	11.39
mmu-miR-193a-3p	63.08	mmu-miR-9-5p	20.24	mmu-miR-34a-5p	11.39
mmu-miR-290a-3p	62.21	mmu-let-7g-3p	20.24	mmu-miR-188-5p	11.39
mmu-miR-582-3p	54.16	mmu-miR-101a-5p	19.02	mmu-miR-218-5p	10.92
mmu-miR-500-3p	48.14	mmu-miR-743a-3p	18.75	mmu-miR-93-5p	10.85
mmu-miR-39-3p	44.3	mmu-miR-875-5p	17.87	mmu-miR-291a-3p	10.77
mmu-miR-882	42.79	mmu-miR-496a-3p	17.38	mmu-miR-532-5p	10.62
mmu-miR-487b-3p	39.92	mmu-miR-181c-5p	17.14	mmu-miR-409-5p	10.48
mmu-miR-874-3p	38.83	mmu-miR-155-5p	16.9	mmu-miR-322-5p	10.4
mmu-miR-375-3p	35.48	mmu-miR-302c-5p	16.79	mmu-miR-327	9.57

mmu-let-7i-3p	34.51	mmu-miR-702-3p	16.44	mmu-miR-700-3p	9.31
mmu-miR-10b-3p	32.88	mmu-miR-142a-3p	16.1	mmu-miR-217-5p	8.81
mmu-miR-411-5p	31.76	mmu-miR-100-5p	15.45	mmu-miR-323-5p	8.39
mmu-miR-300-3p	30.47	mmu-miR-509-5p	15.34	mmu-miR-542-5p	8.33
mmu-miR-294-5p	26.34	mmu-miR-761	15.23	mmu-miR-675-5p	8.33
mmu-miR-743b-5p	25.8	mmu-miR-210-3p	14.82	mmu-miR-1199-5p	8.33
mmu-miR-409-3p	23.9	mmu-miR-343	14.31	mmu-miR-214-3p	8.16
mmu-miR-15a-5p	23.41	mmu-miR-802-5p	13.82	mmu-miR-7a-5p	8.05
mmu-miR-1195	22.3	mmu-miR-19b-3p	13.08	mmu-miR-423-3p	8
mmu-miR-34b-3p	21.1	mmu-miR-678	11.95	mmu-miR-299a-3p	8
mmu-miR-127-5p	20.81	mmu-miR-30b-5p	11.62	mmu-miR-27a-3p	7.94
mmu-miR-547-3p	20.24	mmu-miR-684	11.62	mmu-miR-202-5p	7.94
mmu-miR-804	7.83	mmu-miR-103-3p	5.77	mmu-miR-182-5p	4.82
mmu-miR-202-3p	7.46	mmu-miR-590-5p	5.61	mmu-miR-335-3p	4.82
mmu-miR-497a-5p	7.46	mmu-miR-187-3p	5.77	mmu-miR-130a-3p	4.82
mmu-miR-140-5p	7.21	mmu-miR-467e-5p	5.5	mmu-miR-875-3p	4.79
mmu-miR-744-5p	7.06	mmu-miR-763	5.5	mmu-miR-200a-3p	4.75
mmu-miR-32-5p	7.06	mmu-miR-147-3p	5.46	mmu-miR-707	4.72
mmu-miR-467b-3p	6.86	mmu-miR-30c-5p	5.42	mmu-miR-328-3p	4.69
mmu-miR-181b-5p	6.82	mmu-miR-878-3p	5.35	mmu-miR-33-5p	4.69
mmu-miR-877-5p	6.77	mmu-miR-503-5p	5.35	mmu-miR-20a-5p	4.66
mmu-miR-20b-5p	6.72	mmu-miR-145a-5p	5.35	mmu-miR-291a-5p	4.62
mmu-miR-345-5p	6.58	mmu-miR-434-3p	5.27	mmu-miR-449b	4.62

mmu-miR-450b-3p	6.54	mmu-miR-742-3p	5.24	cel-miR-39-3p	4.62
mmu-miR-681	6.4	mmu-miR-148a-5p	5.24	mmu-miR-338-5p	4.59
mmu-miR-770-5p	6.36	mmu-miR-223-3p	5.2	mmu-miR-141-3p	4.59
mmu-miR-666-3p	6.32	mmu-miR-686	5.13	mmu-miR-29c-3p	4.56
mmu-miR-106a-5p	6.23	mmu-miR-128-3p	5.1	mmu-miR-425-5p	4.47
mmu-miR-654-3p	6.19	mmu-miR-329-3p	5.03	mmu-miR-27b-3p	4.44
mmu-miR-15a-3p	6.19	mmu-miR-453	5.03	mmu-miR-465a-5p	4.4
mmu-miR-139-5p	6.02	mmu-miR-339-5p	4.99	mmu-miR-881-5p	4.37
mmu-miR-501-5p	6.02	mmu-miR-380-5p	4.96	mmu-miR-764-3p	4.34
mmu-miR-183-3p	5.98	mmu-miR-7b-5p	4.92	mmu-miR-384-5p	4.2
mmu-miR-337-3p	5.81	mmu-miR-342-5p	4.89	mmu-let-7g-5p	4.17
mmu-miR-342-3p	5.77	mmu-miR-1188-5p	4.85	mmu-miR-200c-5p	4.17
mmu-miR-208b-3p	4.03	let-7c-5p	-4.40	miR-196a-5p	-2.85
mmu-miR-205-5p	4	miR-719	-4.18	miR-1895	-2.75
mmu-miR-701-5p	3.97	miR-467c-5p	-4.16	let-7d-5p	-2.75
miR-99a-5p	-79.79	miR-720	-3.95	miR-138-5p	-2.72
miR-455-3p	-21.77	miR-883a-3p	-3.79	miR-23a-3p	-2.72
miR-186-3p	-7.98	miR-698-3p	-3.76	miR-146b-5p	-2.70
miR-1187	-7.92	miR-181a-5p	-3.60	miR-295-3p	-2.64
miR-17-3p	-7.78	let-7b-5p	-3.60	miR-574-3p	-2.59
miR-130b-5p	-6.10	let-7e-5p	-3.47	miR-23b-3p	-2.57
miR-296-5p	-5.28	miR-1897-3p	-3.43	miR-146a-5p	-2.56
miR-1897-5p	-4.73	miR-361-5p	-3.34	let-7d-3p	-2.49
miR-1892	-4.53	miR-466c-5p	-3.07		

miR-1194	-4.45	miR-146b-3p	-2.99		

Table S3. Detailed description of the high-fat diet used in the study.

Class description	Ingredient	Grams
Protein	Casein, Lactic, 30Mesh	200
Protein	Cystine, L	3
Carbohydrate	Lodex 10,	125
Carbohydrate	Fine granulated Sucrose	72.8
Fiber	Solka Floc, FCC200	50
Fat	Lard	245
Fat	Soybean oil, USP	25
Mineral	S10026B	50
Vitamin	Choline bitartrate	2
Vitamin	V10001C	1
Dye	Dye Blue FD&C #1, Alum. Lake 35-42%	0.05

Table S4. Primers used in the study

Target	Sequence (5' – 3')	Tm
AhR F	GCAATAGCTACTCCACTTCAG	60.6
AhR R	GGTGTGAAGTCTAGCTTGTG	60.4
VAMP7 F	TCAAGAGCACAGACAGCACTTCC	63
VAMP7 R	GCCATGTAAATCCACCACAGAGAG	61.5
E.coli tnaA F	TGCAACCATCACCAGTAAC	58
E.coli tnaA R	GTCCATTACCACCGGAATATC	60.6
miR-375F	TTTGTTCGTTCGGCTCGCGTGA	64.5

Table S5. List of antibodies used in the study

S. No.	Target	Application	Source	Cat. No.
1	AHR	WB	Santa Cruz	Sc133088
2	VAMP7	IF/WB	Cell Signaling	13876S
3	CD63	IF	Novus Biologicals	NBP2-32830
4	A33	IF/Flow	Biorybt	Orb15687
5	pAkt-1	IF/WB	Cell Signaling	9018S
6	pAkt-2	IF/WB	Cell Signaling	8599S
7	β-actin	WB	Santa Cruz	Sc47778
8	IgG	Pull down	Santa Cruz	Sc65662
9	GAPDH	WB	Santa Cruz	Sc47724

Table S6. List of CRISPR/Cas9 plasmids used in the study

S. No.	Target	Source	Cat. No.
1	VAMP7	Santa Cruz	Sc-423230
